# Hybrid-Protected Perovskite Quantum Dot Films with Ultra-High Efficiency and Stability for LED Backlighting

**DOI:** 10.1021/acsami.4c15012

**Published:** 2024-11-19

**Authors:** Loan Thi Ngo, Wen-Tse Huang, Hemant Verma, Yen-Huei Lin, Ling-Wei Liang, Chia-Te Fang, Jia-Cheng Chang, Wen-Chung Chu, Chaochin Su, Chao-Cheng Kaun, Ru-Shi Liu

**Affiliations:** † Department of Chemistry, National Taiwan University, Taipei 106, Taiwan; ‡ Nano Science and Technology Program, Taiwan International Graduate Program, Academia Sinica and National Taiwan University, Academia Road 128, Nankang, Taipei 115, Taiwan; § Department of Physics, National Taiwan University, Taipei 106, Taiwan; ∥ Research Center for Applied Sciences, Academia Sinica, Academia Road 128, Section 2, Nangang, Taipei 11529, Taiwan; ⊥ Institute of Organic and Polymeric Materials, National Taipei University of Technology, Taipei 106, Taiwan; # Eternal Materials Co., Ltd., Kaohsiung City 821, Taiwan

**Keywords:** perovskite quantum dot film, silicone resin, PMMA, dual-protection methodology, white light-emitting diodes

## Abstract

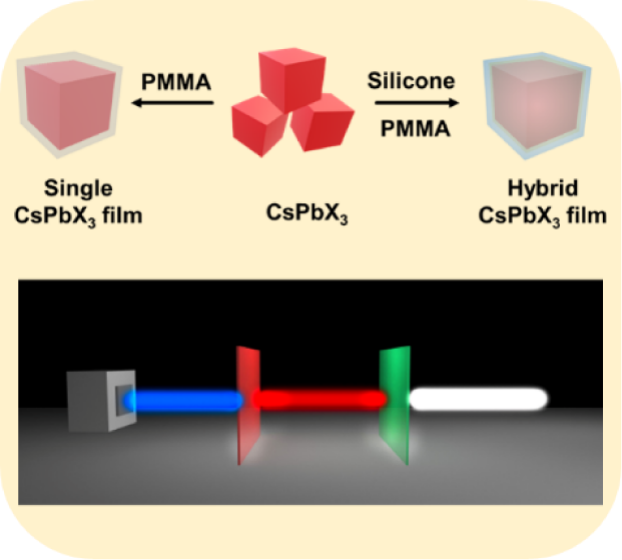

All-inorganic lead halide perovskite quantum dots (PQDs) have emerged as highly promising materials for photonic and optoelectronic devices, solar cells, and photocatalysts. However, PQDs encounter instability and color separation issues because of ion diffusion. Current strategies mainly address stability in green CsPbBr_3_ PQDs, with limited focus on the red-mixed halide PQDs because of their inferior stability compared with green PQDs. Our study provides a new dual-protection methodology for synthesizing high-efficiency green and red mixed-halide PQD films. Red CsPb(Br_0.4_I_0.6_)_3_ and green CsPbBr_3_ PQDs are embedded with silicone resin and then incorporated with poly(methyl methacrylate) (PMMA) matrix to form red and green PQDs@silicone/PMMA films. The high photoluminescence quantum yield (PLQY) and great stability are recorded for the pure-red PQD polymer film. The ultrabright green CsPbBr_3_ PQDs@silicone/PMMA film was also successfully fabricated with an outstanding PLQY beyond 94%. These films exhibited enhanced stability against thermal and environmental degradation, attributed to the dense protective layer of silicone resin and PMMA matrices by the formation of Si–halide and Pb–O bonds, thereby reducing surface defects. Theoretical calculations reveal that combining silicone resin and PMMA improves Pb–O interactions, eliminating uncoordinated Pb^2+^ and enhancing PQD stability. Applied to white light-emitting diodes (WLEDs), these films demonstrated a broad color gamut of 143.4%, indicating their potential for efficient WLED backlighting.

## Introduction

Metal halide perovskite quantum dots (PQDs) are promising semiconducting materials and are described by the general chemical formula ABX_3_ (wherein A is a monovalent cation selected from one or more of Cs, an alkylammonium ion, and a formamidinium ion; B is Pb^2+^ or Sn^2+^; and X is a halogen anion selected from one or more of Br, Cl, or I). This material has demonstrated exceptional optoelectronic properties because of invaluable advantages, such as bright photoluminescence quantum yield (PLQY) with narrow full width at half-maximum (fwhm), adjustable direct bandgaps, significant absorption coefficient, and intrinsically high defect tolerance.^1,2^ Thus, PQDs have garnered growing interest in light-emitting devices (LEDs), solar cells, lasers, and photodetectors. Despite the significant promise demonstrated by electrically driven perovskite LEDs, comparable with organic and quantum dot LEDs in terms of device efficiency, several challenges should be addressed before considering them for commercialization. The primary challenges lie in long-term stability because of their low formation energy, making them susceptible to environmental stresses, moisture, and oxygen and leading to increased defects and reduced carrier lifetimes.^3^ Many passivation strategies have been devised to enhance perovskite stability, but their success has been constrained and achieved to a limited extent. Embedding PQDs in metal oxides, such as SiO_2_ or Al_2_O_3_, faces challenges due to solvent evaporation, resulting in partially exposed perovskites; high-temperature annealing on porous metal oxides leads to substantial decomposition.^4−7^ An alternative approach requires creating composite films by combining perovskite precursors with protective agents, such as polymers, organic small molecules, or inorganic nanoparticles. Although this method is straightforward, it frequently leads to significant phase separation between perovskites and the protective media, resulting in broadening photoluminescence (PL) peaks, reduced PLQY, and inadequate protection.^3^ In addition, almost all current passivation methods have used green APbBr_3_ PQDs (CsPbBr_3_ and MAPbBr_3_) as a primary target. This substance exhibits significantly greater stability than red PQDs, particularly those incorporating mixed halides.^2,8−10^ Currently, mixed-halide perovskites, such as APbBr*_x_*I_3–*x*_, have been utilized to fabricate pure-red thin-film PQDs LEDs (emission from 640–670 nm).^11−13^ However, these devices have frequently demonstrated suboptimal performance with poor stability and low PLQY due to severe phase segregation, increased ionic diffusion, and thermal instability, resulting in low efficiency and early decomposition.^11,14−17^ All-inorganic red CsPbI_3_ perovskites, which are known for their enhanced thermal stability, emerge as a promising candidate for creating red-emission perovskite LEDs with stability and high efficiency.^13,18,19^ Nevertheless, CsPbI_3_ thin films face difficulties concerning phase stability and deep-red region.^11,20^ Thus, addressing these challenges and enhancing the efficiency and stability of pure-red mixed halide perovskite are crucial steps in advancing the technology toward practical commercial applications.

Silicone resins, known for their remarkable versatility and safety, have found widespread applications in industries and daily life and have been used to enhance the water and thermal resistance of diverse PQDs because of their outstanding water-repellency and heat-resistant properties.^6,21,22^ In detail, the water, light, and heat resistance of green MAPbBr_3_ nanocrystals significantly increased with the formation of a nanofluid with various silicone oils.^6,23^ Moreover, a range of CsPbX_3_-silicone resins were fabricated using silicone resin as a precursor. The composite of CsPbBr_3_-silicone resin displays outstanding stability against heat and moisture and maintains PL emission after one year in open air or two months in water because of the protective properties of the silicone resin.^24^ Thus, silicone resin is a promising candidate for stabilizing red mixed halide perovskites.

In our study, we developed a comprehensive methodology for producing highly efficient and stable pure red mixed-halide perovskite film by blending CsPb(Br_0.4_I_0.6_)_3_ PQDs within silicone resin and poly(methyl methacrylate) (PMMA) polymer matrices at room temperature (RT) to obtain high PLQY (above 43%). The method was also successfully applied to form green CsPbBr_3_ PQD film with ultra stability and exceptional PLQY (above 94%). The red CsPb(Br_0.4_I_0.6_)_3_ PQD film remains stable after prolonged air exposure and shows excellent thermal stability, with a slight increase in PL intensity following thermal cycling. Experimental and theoretical investigations were carried out to elucidate the efficiency of our protective strategy. The results indicate that incorporating both silicone resin and PMMA into the perovskite greatly strengthens the Pb–O interaction, compared to using either silicone resin or PMMA individually. In addition, forming Pb–O and Si–halide effectively hinders the diffusion and self-release of halide ions and eliminates the uncoordinated Pb. The resultant perovskite films, including red and green PQD films, were employed in white light-emitting devices (WLED), which exhibited an expansive color gamut, reaching 143.4% of the National Television System Committee (NTSC). This work proposes a novel and easily scalable method for the preparation of PQD film to advance its commercial utilization in LEDs.

## Results and Discussion

### Film Preparation

**Scheme 1:**
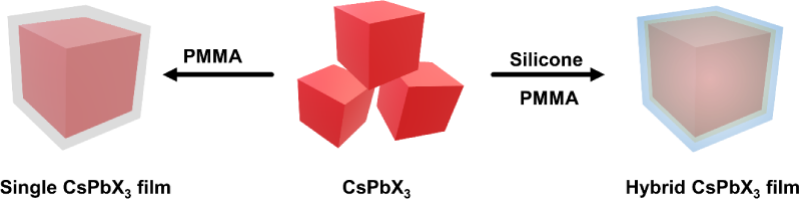
Preparation of CsPbX_3_ PQD Film by Dual Encapsulation with Silicone Resin and PMMA

Two kinds of films are fabricated in our study (Scheme 1). Herein, a single PQD film (SP film) is formed by encapsulating PQDs with only PMMA (PQDs/PMMA), while the hybrid PQD film (HP film) is fabricated by mixing PQDs with silicone resin and PMMA (PQDs@silicone/PMMA). In detail, the HP film is formed first from the synthesis of high quantum yield PQDs by using our microfluidic system. Afterward, the as-formed PQD solution is placed in a vacuum to remove the hexane solvent. As evidenced in earlier studies, silicone resin achieves high dispersion, effective coordination, and defect healing in PQDs, attributed to its compact Si–O–Si units, which create Si–I and Pb–O bonds with the perovskite surface.^21,22^ Thus, the as-dried PQDs are then mixed with the silicone resin until the homogeneous state of the PQDs@silicone composite is obtained. This step aims to form the first protective layer for PQDs and to protect them from oxygen and moisture in the surrounding environment. As our observation, this composite can last for months without quality degradation. However, the mixture of PQDs and silicone resin can only be dried at high temperatures (150 °C) for 2–3 h. Moreover, the high quantum efficiency of the PQD film is difficult to obtain after heating because of the instability of PQDs at high temperatures. Thus, a suitable agent should be identified for the synthesis of PQDs@silicone composite, which can be dried at lower temperatures. According to our density functional theory (DFT) calculations, the interaction between Pb and O is greatly improved by simultaneously incorporating silicone resin and PMMA into perovskite, as opposed to adding just one of them. As a result, the mixture is further blended with the PMMA polymer, which is dissolved in toluene for the optimal time. PMMA not only involves the solidification of the PQDs@silicone mixture at RT to yield the HP film but also provides the second protective layer for the PQDs. A SP film is also fabricated to compare with the same protocol without introducing silicone resin in the film.

### Film Characterization

**Figure 1:**
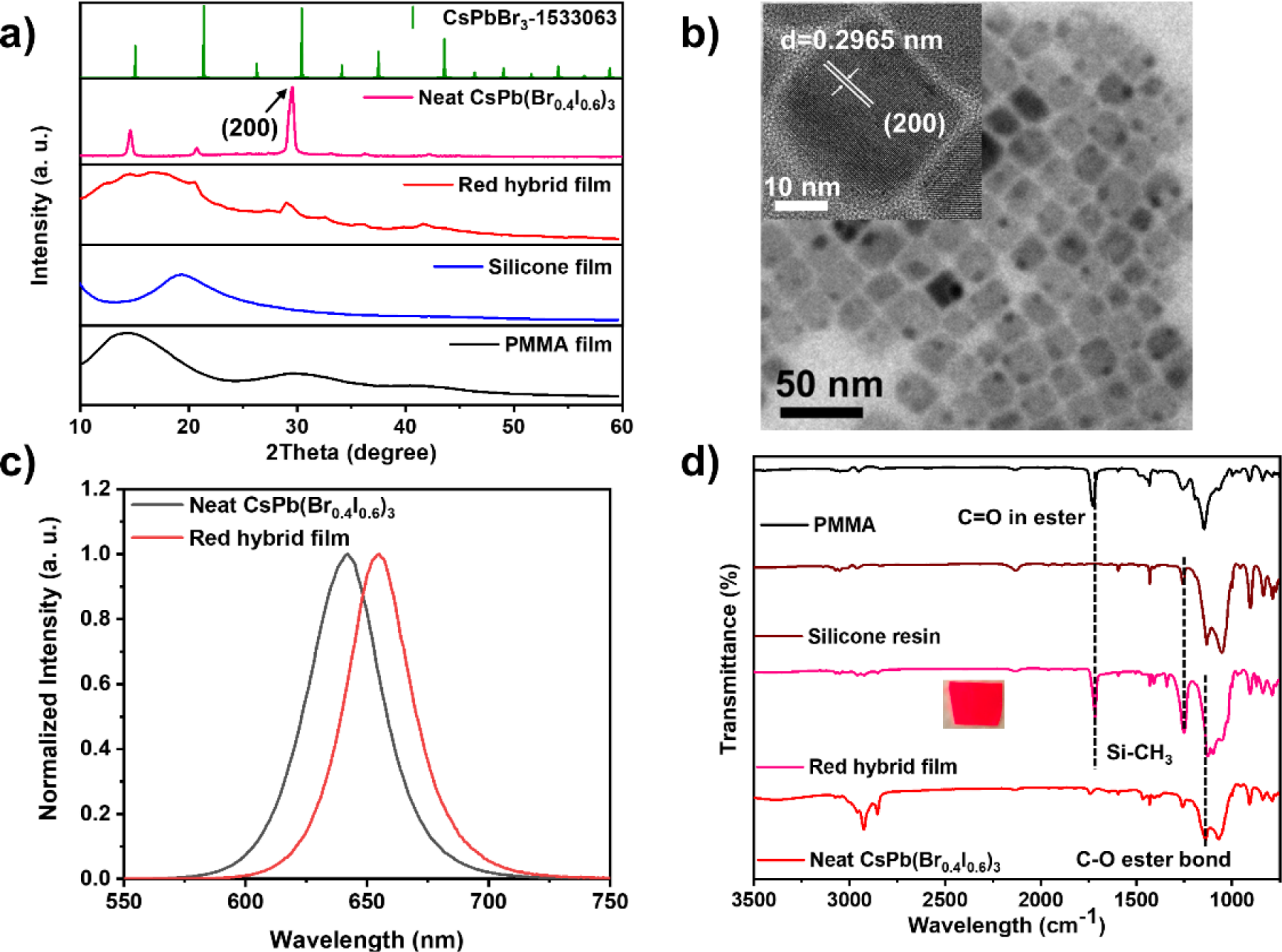
RHP film characterization. (a) XRD patterns. (b) TEM images of CsPb(Br_0.4_I_0.6_)_3_ PQDs. Inset: high-resolution TEM image of CsPb(Br_0.4_I_0.6_)_3_ PQDs. (c) PL spectra of neat CsPb(Br_0.4_I_0.6_)_3_ PQDs and RHP film. (d) FTIR spectra of neat CsPb(Br_0.4_I_0.6_)_3_ PQDs and the RHP film.

Aiming to illustrate the effectiveness of our strategies, both pure red CsPb(Br_0.4_I_0.6_)_3_ films and green CsPbBr_3_ films were produced and investigated. For red CsPb(Br_0.4_I_0.6_)_3_ films, as mentioned in Scheme 1, two types of films, namely, red CsPb(Br_0.4_I_0.6_)_3_@silicone/PMMA film (RHP film: red hybrid film) and red CsPb(Br_0.4_I_0.6_)_3_@PMMA film (RSP film: red single film), are fabricated for comparison. Note that the CsPb(Br_0.4_I_0.6_)_3_ PQDs experience rapid degradation within a few minutes of the introduction of PMMA (Video S1) due to the formation of the CsPb(Br_0.4_I_0.6_)_3_ black phase.^11,25^ Thus, we did not further perform the characterization of the RSP film. The RHP film characterization is presented in Figure 1. Figure 1a depicts the X-ray diffraction (XRD) analysis of the RHP film. The broad peaks from 10° to 20° are ascribed to the peaks of silicone resin and PMMA films. Other peaks from the RHP film also appear in the CsPb(Br_0.4_I_0.6_)_3_ PQDs and match with the standard XRD patterns of cubic-phase CsPbBr_3_ (COD-1533063). Phase transfer of CsPb(Br_0.4_I_0.6_)_3_ PQDs does not occur after forming the film. The morphology and particle sizes of the obtained PQDs are examined using a transmission electron microscope (TEM), revealing a cubic shape with an average size of 15.2 ± 2.5 nm (Figure 1b). The high-resolution TEM (HRTEM) micrograph confirms the high crystallinity of CsPb(Br_0.4_I_0.6_)_3_ PQDs, as evidenced by the planar spacing of 2.97 Å, corresponding to the (200) plane (Figure 1b, inset).

The PL properties of the RHP film are investigated using a PL spectrometer. In comparison with the CsPb(Br_0.4_I_0.6_)_3_ PQDs, a red shift is observed after forming the film (Figure 1c). For the red CsPb(Br_0.4_I_0.6_)_3_ PQDs, the emission center is approximately 641 nm; after forming the RHP film, the PL emission is around 654 nm with the 450 nm excitation. The phenomenon can be explained by the quantum confinement caused by changing the surface environment of the quantum dots. In addition, the passivation of surface defects on the PeQDs by silicone/PMMA encapsulation reduces nonradiative recombination pathways and leads to a slightly narrower bandgap, also manifesting as a redshift in the PL emission.^26^ Finally, the pure PMMA film, silicone resin film, and RHP film are compared using the Fourier-transform infrared spectroscopy (FTIR) spectra. As shown in Figure 1d, the prominent peaks of PMMA and silicone resin are presented in the RHP film. In detail, the ester C=O group at 1731 cm^–1^ and the C–O ester bond at 1150–1000 cm^–1^ of PMMA appear in the FTIR spectra of PMMA film and RHP film.^27^ Additional peaks at 1260 cm^–1^ associated with the stretching vibration of Si–CH_3_ are evident in the FTIR spectra of the silicone and RHP films.^28^ This finding confirms that the silicone resin and PMMA polymer successfully encapsulated the CsPb(Br_0.4_I_0.6_)_3_ PQDs.

For green CsPbBr_3_ films, the green CsPbBr_3_@silicone/PMMA film (GHP film: green hybrid film) and the control CsPbBr_3_@PMMA film (GSP fim: green single film), which provide bright luminescence after forming the green films, were also produced and examined. In this regard, green films are characterized. The detailed characterization results of the GHP and GSP films are presented in Supporting Information (Figure S1 and Tables S1 and S2). Briefly, the PL redshift is more severe in the GSP film than in the GHP film and CsPbBr_3_ due to the direct exposure of PQDs to PMMA polymer, leading to serious aggregation. By contrast, the first silicone resin protective layer facilitates PQD dispersion in the GHP film, resulting in a minor redshift. As shown in Table S2 and Figure S1c, the GHP film has a remarkably longer lifetime than the GSP film and neat CsPbBr_3_ PQDs. The extension of the lifetime implies a reduction in the decay rate of excitons, suggesting that the surface traps of CsPbBr_3_ PQDs may be mitigated by silicone resin and PMMA polymer matrices.^6^ The exceptional PLQY is seen in the GHP film (94.17%), while the PLQY is only 74.21% in the GSP film (Table S1). These findings clearly demonstrate that the performance of the film with the dual protection of silicone and PMMA is significantly enhanced compared with CsPbBr_3_ PQDs and single PMMA-protected CsPbBr_3_ film.

### Effects of Silicone Resin and PMMA Ratios on the Films

**Figure 2:**
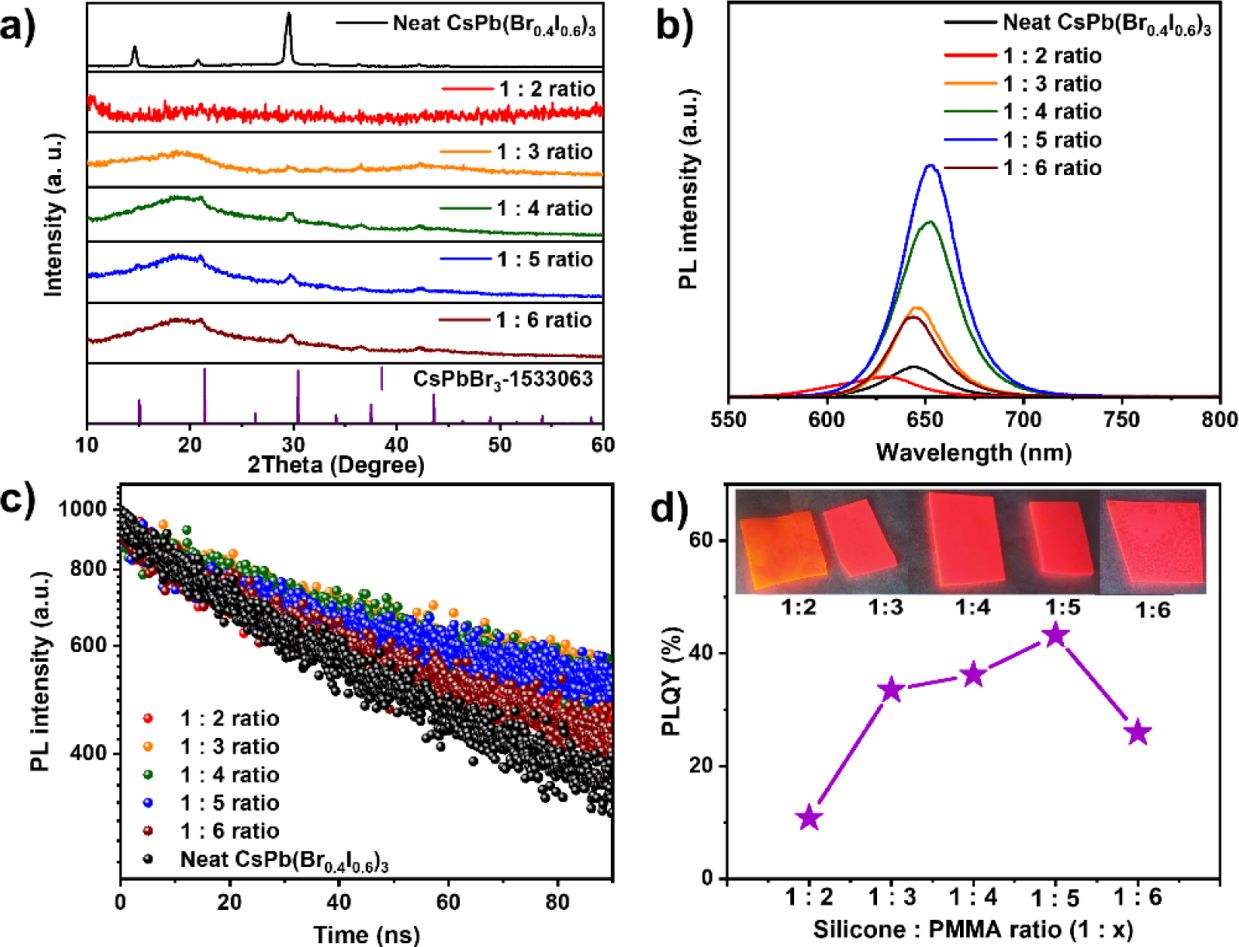
Effects of silicone resin and PMMA ratios on the films. (a) XRD, (b) PL spectra, (c) PL decay curves, and (d) PLQY comparison of RHP film encapsulated with different ratios of silicone and PMMA.

The amount of PMMA and silicone plays an imperative role in determining the solidification and luminescence of the film. A series of films with various ratios of silicone resin: PMMA (by weight) is developed and used to examine the effects of silicone resin and PMMA on the RHP film. The RHP film could not be successfully formed at 1:1 ratio. Thus, the silicone resin and PMMA ratio is set from 1:2 to 1:6. XRD, PL, lifetime, and PLQY values are used to evaluate the quality of the formed film to establish the optimal condition for the film. As shown in Figure 2a, at a 1:2 ratio, the PQDs in the film are rapidly damaged after being exposed to the air since the film is not thoroughly dried at the examined time. Thus, the phase of CsPb(Br_0.4_I_0.6_)_3_ PQDs in this film could not be seen. The RHP film with ratios from 1:3 to 1:6 also shows a single phase. In addition to XRD, we further analyze the PL intensity, lifetime, and PLQY of the films at these specific ratios in order to confirm the optimal ratio for the RHP film.

As depicted in Figure 2b,c and Table S3, the PL intensity and PL lifetime increase with increasing PMMA concentration. These phenomena are ascribed to the improved film quality and decreased nonradiative recombination. In addition, the PL emission of 1:2 shows a blue shift compared to the neat PQDs and other samples. This can be attributed to the rapid degradation of CsPb(Br_0.4_I_0.6_)_3_ PQDs in the ambient atmosphere during PL measurement once the film is not completely dried. This is also consistent with the XRD results. The optimal ratio is at 1:5. The decrease in the quality of the RHP film at the ratio of 1:6 is due to the aggregation quenching caused by the excess of PMMA with more polar functional groups. This phenomenon is consistent with the rapid quenching of the red CsPb(Br_0.4_I_0.6_)_3_ PQDs after introducing the PMMA solution into the CsPb(Br_0.4_I_0.6_)_3_ PQDs. Moreover, Figure 2d and Table S3 show that the RHP film achieves a maximum PLQY of up to 43.21%) at a ratio of 1:5, which is an excellent result for pure-red PQD film to date (Table S4).

### Stability Test

**Figure 3:**
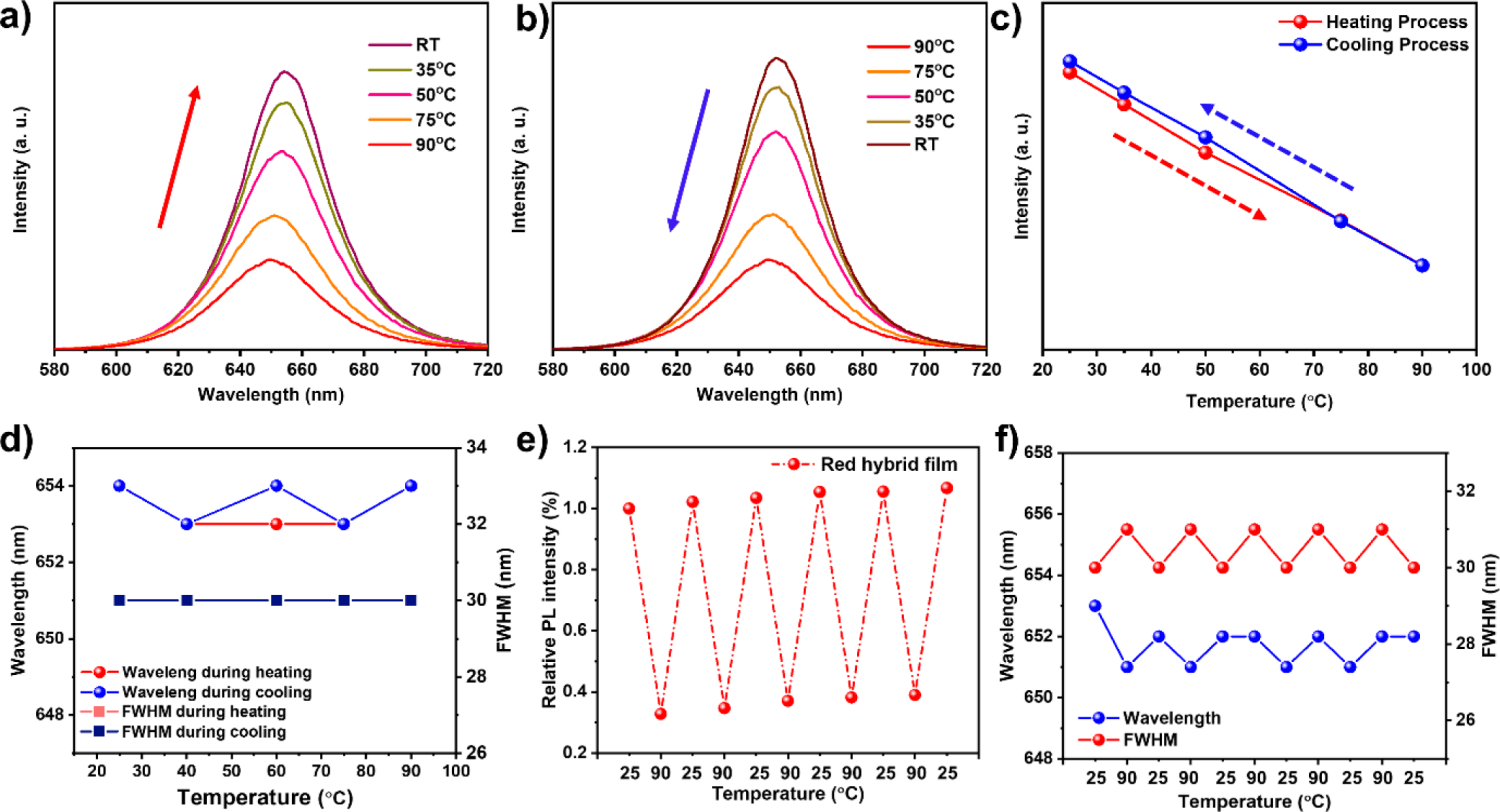
Thermal cycling of RHP film with (a) heating and (b) cooling. Comparison of (c) the PL intensity change, (d) wavelength and fwhm of RHP film during heating and cooling procedures. (e) PL intensity of the RHP film after undergoing five cycles of heating and cooling between 25 and 90 °C, and (f) PL emission peaks and fwhm of the RHP film after five rounds of heating/cooling processes.

Achieving stability is a pressing challenge that must be addressed to commercialize PQD films successfully. The stability assessment of PQD films is conducted from three distinct perspectives, including thermal cycling, multithermal cycling, and air exposure, with neat PQDs serving as reference materials. The thermal cycling test of the films is evaluated by subjecting them to elevated temperatures and then cooling them back to RT. As shown in Figure 3a–c, the RHP film reveals a remarkable recovery of the initial PL intensity, reaching 104% of the initial emission intensity. As well-known, when the temperature increases, thermal effects can cause changes in the material’s electronic structure, leading to a decrease in PL intensity. However, when the temperature is lowered during cooling, the material can return to its original state, restoring or even slightly enhancing the PL intensity. Herein, the slight increase to 104% could be due to minor structural relaxation and improved ordering at the lower temperature, which enhances the emission efficiency slightly beyond its original value. This behavior suggests that the material maintains its structural and optical integrity through thermal cycling, indicating good thermal stability. Furthermore, the wavelength and fwhm change during heating and cooling are investigated (Figure 3d). The film returns to the exact initial PL emission peak with the same fwhm throughout the thermal cycle, which could be attributed to the exceptional protective capacity of the dual protective layer of silicone and PMMA on the CsPb(Br_0.4_I_0.6_)_3_ PQDs. To further confirm the thermal stability of the HP films, five rounds of heating and cooling processes are performed. No obvious changes are observed in the PL intensity between 25 and 90 °C (Figure 3e). The increase in PL intensity between rounds can be ascribed to the slow surface passivation of the hybrid layer of silicone and PMMA on the PQDs. The PL emission peak and fwhm can also return to the initial value in the range of 25 to 90 °C (Figure 3f), proving the outstanding thermal stability of the RHP film. For air exposure, after over 21 days, the PL intensities of the RHP film remain 100% of the initial one, while the neat CsPb(Br_0.4_I_0.6_)_3_ PQDs remain only 81.76% of the initial one (Figure S2a). We also performed another thermal test at 90 °C. We saw that after continuous heating, the PL intensity of the RHP film will continue to rise (Figure S2b). After 50 h, the PL intensity can reach 140% of the initial PL intensity. This is well-matched with the results of multiple-thermal cycling. These findings confirm that the dual-matrix encapsulation of the PQD film with silicone resin and PMMA polymer guarantees the prolonged stability of QPD films. The enhanced stability and reversibility can be attributed to the slow surface passivation, effective inhibition of undesirable grain growth, and heat-induced aggregation of PQDs.^29^

The stability test is also performed for the GHP film, with the GSP film and neat CsPbBr_3_ PQD counterparts as references. The GHP film exhibits enhanced stability compared with the GSP film and neat CsPbBr_3_. The detailed explanation is presented in Figures S3 and S4. In summary, for the GSP film, the thermal test was carried out from 25 to 90 °C. The PL intensity of the GHP film approaches 99% of the initial PL intensity in the thermal cycling test without changes in PL emission and fwhm after heating and cooling cycling (Figure S3a–c). The outstanding thermal reversibility of the GHP film is also seen after multiple heating/cooling processes with negligible change in the PL intensity, PL emission peaks, and fwhm (Figure S3e,f). After five rounds of heating and cooling, the PL intensity of the fifth round still keeps at 99% compared to that of the first one. Moreover, after 50 days of air aging, the PL intensity remains at 100% PL intensity compared to the first one (Figure S4a). Moreover, in line with earlier findings, the luminescence degradation of CsPbBr_3_@PMMA occurs at temperatures exceeding 80 °C due to the PMMA glass transition temperature (105 °C).^30^ A thermal test is thus conducted at 80 °C to evaluate the effects of silicone. The significant difference between the GHP film, the GSP film, and the neat CsPbBr_3_ PQDs is seen after heating them at 80 °C. The GHP film remained at 100% of the initial value of PL after 17 days, the GSP film remained at only 18.57% of the initial one after 17 days, and the neat CsPbBr_3_ PQDs completely lost the PL intensity after 17 days. After 50 days of heating, the GHP film still maintains 70.29% of the initial value (Figure S4b). As a result, after the introduction of the silicone resin into the CsPbBr_3_@PMMA, the thermal stability is enhanced. This finding implies that the silicone resin and PMMA polymer matrices provide enhanced protection for the structure of CsPbBr_3_ PQDs.

### Structure Investigation

**Figure 4:**
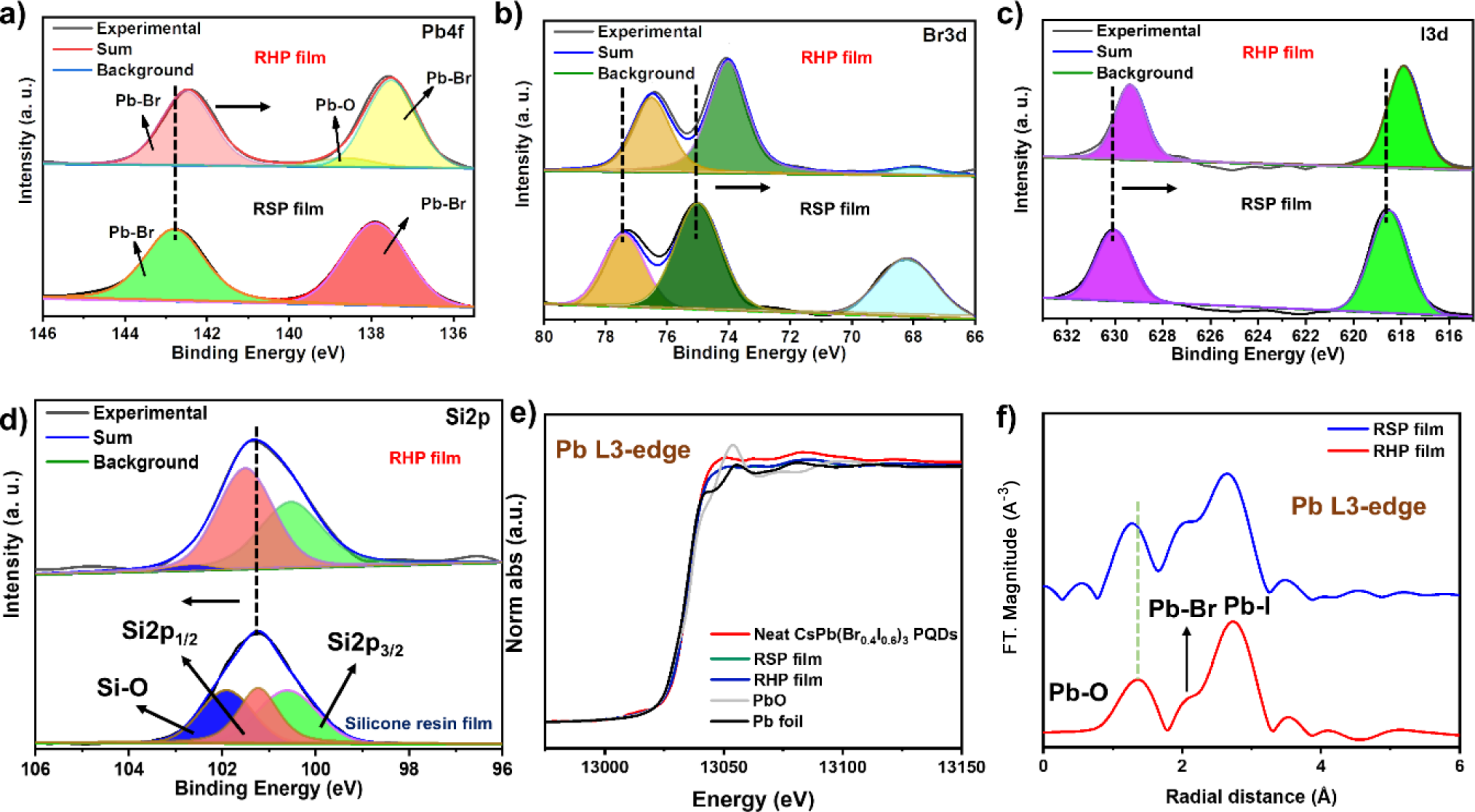
Comparison of the RHP film and the RSP film (a) Pb 4f, (b) Br 3d, (c) I 3d, (d) Si 2p, (e) XANES spectra, and (f) Fourier-transformed EXAFS spectra.

The surface structure of the PQDs in the film was investigated to understand the reasons behind the stability improvement of PQDs after forming the RHP film. From the film elemental survey, we can see the presence of all the elements, including Cs, Pb, Br, I, C, O, and Si (Figure S5a). In the X-ray photoelectron spectroscopy (XPS) survey of RSP film, Cs, Pb, Br, I, C, and O elements are shown without the presence of Si element because of the absence of the silicone resin (Figure S6a). The detailed Cs 3d, Pb 4f, Br 3d, I 3d, C 1s, O 1s, and Si 2p high-resolution XPS of the RHP and RSP films can be found in Figures S5b–h and S6b–g, respectively. Herein, the RHP and RSP films are compared to confirm the essential difference between the films with and without silicone (Figure 4). The RHP film modified with silicone resin exhibits a coordination interaction that causes shifts in Pb 4f, Br 3d, and I 3d toward lower binding energies in comparison with those in the control RSP film (Figure 4a–c).^21^ The Si 2p XPS spectrum underwent deconvolution into three peaks in both the RHP film and the silicone resin film. However, the percentage of Si–O in the RHP film is only smaller than that of the silicone resin film. In addition, the Si 2p peak shifts to a higher energy binding than the pure silicone resin film (Figure 4d). This finding could be attributed to the interaction of halide vacancies of PQDs and Si in the silicone resin to form halide–Si bonds.^22^ The binding energy of metal oxide also appears in O 1s (529.2 eV) (Figure S5g) and Pb 4f (138.5 eV) (Figure S5c) in the RHP film. The appearance of these peaks indicates that the combination between CsPb(Br_0.4_I_0.6_)_3_ PQDs and silicon resin@PMMA mixture forms the Pb–O bond. In the previous report, the silicone resin network forms Si–O–Si bonding, establishing the Pb–O bond with the perovskite, achieving effective defect passivation along with iodine immobilization.^21^ Small amount of Pb–O can be seen in RHP film, while the Pb–O is not obviously seen in the RSP film. However, a previous study illustrated that the chelation between C=O and Pb^2+^ ions occurs after the introduction of PMMA to PQDs and enhances the thermal stability of the film.^31^ Thus, we further performed X-ray absorption spectroscopy (XANES) measurements and the extended X-ray absorption fine structure (EXAFS) analysis (the Pb *L*_3_-edge) to confirm the differences between the two films and the existence of the Pb–O bond in the RSP film. Herein, the K-edge of Pb was examined using beamline TPS 44A at the Center of National Synchrotron Radiation Research, Taiwan. As shown in Figure 4e, the oxidation state of the two films is at the same position with a negligible shift compared with the neat CsPb(Br_0.4_I_0.6_)_3_ PQDs. This phenomenon arises from changes in the surrounding environment caused by the interaction of polymers with PQDs. In the EXAFS profile, two bonds, namely, Pb–O and Pb–Br, can be observed (Figure 4f). The intensity of the Pb–I bond is higher in the RHP film than in the RSP film. This finding is ascribed to the higher Pb–I bond length disorder in the RSP film compared with the HP film.^32^ In addition, Pb–O appears in both films, pointing out that Pb–O bonds are formed from the silicone resin and PMMA. The RSP film exhibits a reduction in the average Pb–O and Pb–I bond lengths compared with the HP film, suggesting lattice compression.^33^ This finding could be attributed to the interaction of the silicone resin and PMMA with the PQD surface and is consistent with the XPS results obtained from the HP film.

We also conduct an XPS comparison between the film and the GSP film (Figures S7–S9). A detailed explanation can be found in Supporting Information. The significant change in Pb 4f, Br 3d, and Si 2p from the GHP film to the GSP film is assigned to the formation of Pb–O and Si–Br between the silicone network and CsPbBr_3_. This result matches the phenomenon observed in the RHP film.^21^ Moreover, the Pb–O formation can be found in the GSP and GHP films, which can explain the effective encapsulation of PMMA in both films.

### Theoretical Calculation

**Figure 5:**
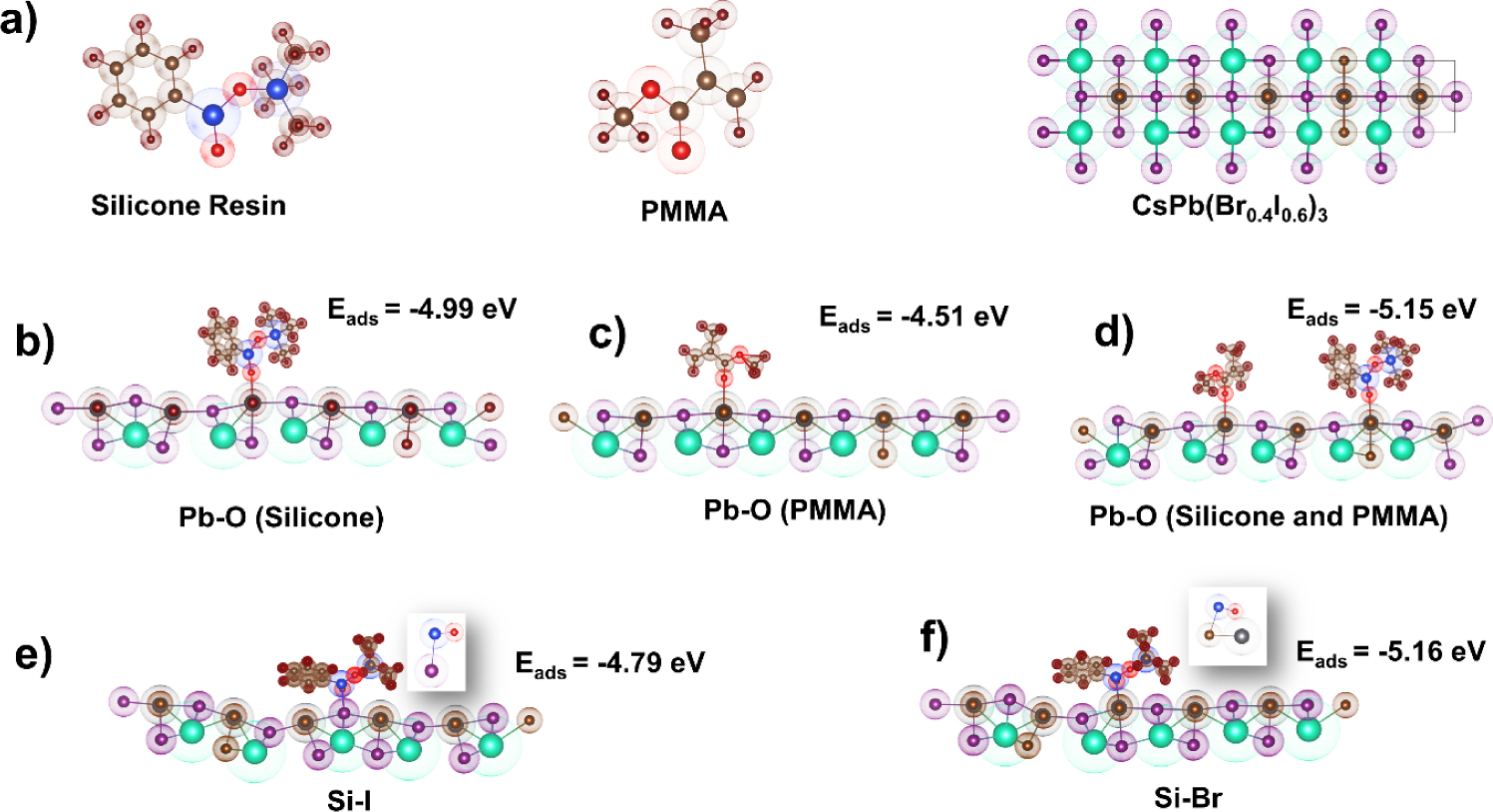
(a) Optimized structures of silicon resin, PMMA, and CsPb(Br_0.4_I_0.6_)_3_ perovskite. Interaction between CsPb(Br_0.4_I_0.6_)_3_ perovskite and (b) silicone resin with the adsorption site of Pb–O, (c) PMMA with the adsorption site of Pb–O, and (d) both PMMA and silicone resin with the adsorption site of Pb–O. Interaction between PQDs and silicone resin with the adsorption site of (e) Si–I and (f) Si–Br.

Density functional theory (DFT) is performed to determine the adsorption energy (*E*_ads_) of different polymers on the CsPb(Br_0.4_I_0.6_)_3_ perovskite surface, validating our hypothesis regarding the use of a PMMA and silicone blend and elucidating the stability enhancement mechanism of the RHP film, along with the interaction dynamics between the red perovskite and the polymers (silicone resin and PMMA). The optimized lattice structure of the 1 × 1 × 5 supercell CsPb(Br_0.4_I_0.6_)_3_ and silicone resin and PMMA polymers is depicted in Figure 5a.^34^ The interaction of silicone and perovskite can be caused by the interaction of −Si–O–Si– and the uncoordinated Pb^2+^ to form Pb–O. The chelation between −C=O of PMMA and Pb^2+^ can form Pb–O. Thus, the adsorption energies for the Pb–O sites are calculated based on the interaction of the perovskite–silicone resin, perovskite–PMMA, and perovskite–silicone resin/PMMA. As shown in Figures 5 and S10, the *E*_ads_ values at −4.99 eV (Figure 5b) and −4.51 eV (Figure 5c) are recorded for the perovskite–silicone resin and the perovskite–PMMA, respectively. Interestingly, when silicone resin and PMMA are incorporated simultaneously, the adsorption energy (−5.15 eV) significantly changes (Figure 5d). Our calculations indicate that the interaction between Pb and O becomes significantly more favorable when silicone resin and PMMA are simultaneously introduced into perovskite compared with solely incorporating either silicone resin or PMMA into perovskite. Additionally, we investigate the secondary interaction between Si and halides, which has been proposed in earlier studies.^21^ The *E*_ads_ values are −4.79 eV for Si–I (Figure 5e) and −5.16 eV for Si–Br (Figure 5f). The large difference between these adsorption energies could be attributed to the cumulative effect of the Pb–O interaction with the Si–Br (inset Images, Figure 5e,f). The favorable energy configurations of Si–I and Si–Br illustrate the robust interaction between Si–I and Si–Br that could potentially inhibit the diffusion of halides within the perovskite, thereby contributing to an additional increase in stability.^21^

Overall, we suggest that the first layer of silicone resin uniformly encapsulates the entire perovskite surface by forming Pb–O and Si–halide bonds, thereby reducing halide diffusion (I and Br) and effectively passivating the surface defects.^21,35^ Moreover, the left vacancies of uncoordinated Pb atoms can be occupied by forming Pb–O between the second protective layer of PMMA and PQDs, resulting in a compact protective layer, which enhances the stability of the PQD film.

### Optical Density

Optical density typically refers to the logarithmic ratio of incident light to transmitted light through a material. It is often used in the examination of blue light absorption. The absorption of blue light by materials is critical for the efficient operation of LEDs because it enables the creation of white light. High blue light absorption is crucial for LED efficiency.

Figure S11 shows the blue light absorption with the corresponding emissions of the RHP film compared with the red phosphor KSF@silicone@PMMA film (red hybrid KSF film). As shown in Figure S11a and Table S5, higher blue light absorption is observed for the RHP film than for the red hybrid KSF film, excited by an emitting light of about 450 nm. The single emission peak with narrow fwhm is also seen in the RHP film; in the phosphor hybrid KSF film, various emission peaks are observed (Figure S11b). This finding indicates that the RHP film possesses a better pure quality of color. A comparison of blue light absorption is also performed for GHP and GSP films and green phosphor beta-SiAlON: Eu^2+^ film (Figure S12 and Table S5). The GHP film has one peak of emission and narrower fwhm compared with the green phosphor beta-SiAlON: Eu^2+^ film that compensates for the slightly lower optical density value of GHP film to green hybrid beta-SiAlON: Eu^2+^ film. The GHP film also possesses better optical density than the GSP film. As a result, the red and green HP films are both promising materials for LEDs.

### Optical Characteristics of All-Inorganic PQD Films in White Light Emitting Diode (WLED)

**Figure 6:**
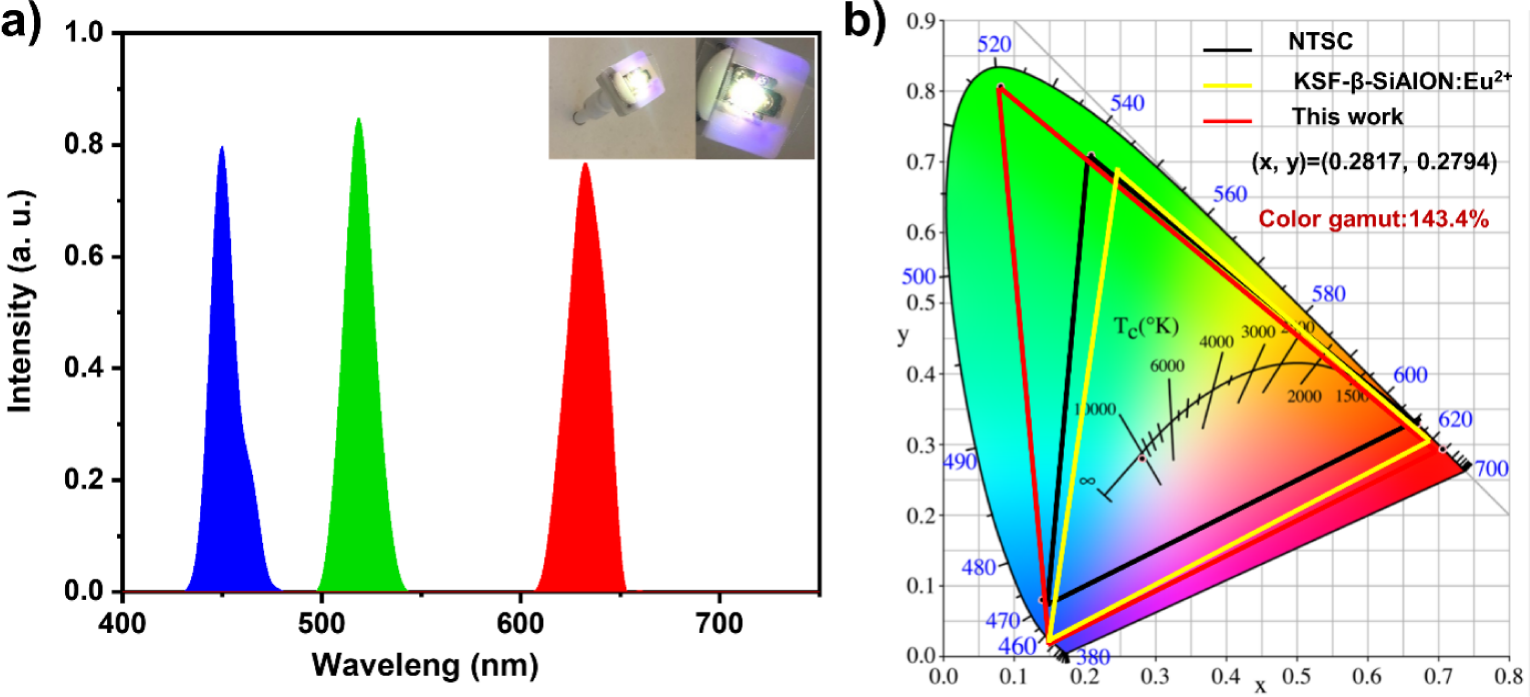
(a) PL spectra of the WLED were fabricated by using red and green HP films. The inset in the upper left corner shows an image of the WLED operating at 2.8 V and 20 mA. (b) CIE chromaticity coordinates and color gamut of the WLED.

Figure 6 illustrates the optical characteristics of our RHP and GHP films in WLEDs. The PL spectra for the WLED were generated using HP films (Figure 6a). The comparison of the color gamut areas of WLEDs based on PQD films and commercial phosphors (beta-SiAlON and KSF) was depicted in Figure 6b. At a current of 20 mA, our operational WLED (inset of Figure 6a) demonstrated impressive optical characteristics, with a good luminous efficacy of 229 lm/W and an excellent color rendering index of 99.73. The WLED’s chromaticity coordinates were (0.2817, 0.2794), corresponding to a correlated color temperature of 9824 K. The RGB chromaticity coordinates stand at (0.7062, 0.2937), (0.0813, 0.8063), and (0.1534, 0.0212). The color gamut, enclosed by the triangle on the CIE1931 chromaticity diagram, encompasses approximately 143.4% of NTSC. This performance greatly surpasses that of beta-SiAlON: Eu^2+^ green phosphors and KSF red phosphors-based WLED (which achieves 113% of NTSC). This finding suggests the high potential of all-inorganic PQD films embedded in silicone resin and PMMA polymer matrix for color conversion applications.

## Conclusion

We have successfully synthesized and produced high-efficiency red and green PQDs@silicone/PMMA films. The dual-protection methodology involved the initial embedding of PQDs with silicone resin to form a PQDs@silicone composite, followed by the incorporation of this mixture into the PMMA matrix to form the HP film at RT. The study successfully produced a red mixed-halide CsPb(Br_0.4_I_0.6_)_3_ film with PLQY surpassing 43% and good optical density. The ultrabright GHP film also exhibited exceptional PLQY exceeding 94%. The HP films demonstrated enhanced stability against thermal, moisture, and oxygen degradation in comparison with their respective PQD solutions and SP film. The stability improvement of the HP film can be explained by fabricating the denser protection layer of silicone resin and PMMA matrices on the PQD surface, which reduced the surface defects of the PQDs compared with the film without silicone resin and PQD solution. Additionally, the WLED device utilizing our RHP and GHP films demonstrated a broad color gamut of 143.4% NTSC. As a result, the PQDs films containing PQDs encapsulated in silicone resin and PMMA matrices demonstrate potential applications as efficient color-converting materials for WLEDs and backlighting.

## Theoretical Calculations

Density functional theory (DFT) calculations were used to explore the adsorption energy of different polymers on the surface of CsPb(Br_0.4_I_0.6_)_3_ nanocrystals. In our study, all computational analyses were conducted employing the *Vienna Ab initio Simulation Package* (VASP)^36,37^ within the projector augmented wave (PAW)^38^ approach and generalized gradient approximation (GGA)^39^ in the Perdew–Burke–Ernzerhof (PBE) exchange–correlation function.^40^ A cutoff energy of 500 eV was utilized in the solution of the Kohn–Sham equation with a plane wave basis. A 1 × 1 × 1 gamma centered mesh^41^ was used to sample the Brillouin zone. All geometric structures were relaxed until the electronic energy and force components were below 1.0 × 10^–6^ eV and 0.02 eV/Å, respectively.

The equation below was used to compute the absorption energies of silicone resin and PMMA on the perovskite surface.
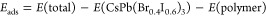
1where *E*_ads_ represents the amount of energy absorbed, *E*(total) is the overall energies of the supercell with silicone resin and PMMA on the perovskite substrate, *E*(CsPb(Br_0.4_I_0.6_)_3_) is the perovskite substrate only, and *E*(polymer) is the energy of the silicone resin or PMMA.

## Experimental Section

### Materials

Cesium carbonate (Cs_2_CO_3_, 99.99%), oleic acid (OA, 90%), octadecene (ODE, 90%), lead(II) bromide (PbBr_2_, 99.99%), and lead(II) iodide were provided by Thermo Scientific. Zinc bromide was obtained from Alfa Aesar. Toluene (99%) and hexane (95%) were acquired from Echo. Oleylamine (OAm, 80–90%) was purchased from Showa. Silicone resins A and B (OE6631) were supplied by Dow Corning Co. Poly(methyl methacrylate) (Mw = 996000 g/mol) was bought from Aldrich. K_2_SiF_6_:Mn^4+^ (KSF) and green beta-SiAlON:Eu^2**+**^ were provided by Everlight company.

### Synthesis of All-Inorganic Lead Halide Perovskite Quantum Dots by Using a Microfluidic System

#### Synthesis of CsPb(Br_0.4_I_0.6_)_3_

In a 100 mL three-necked bottle, 0.165 g of Cs_2_CO_3_, 45 mL of octadecene (ODE), and 5.7 mL of oleic acid (OA) were dehydrated under vacuum conditions at 120 °C for 1 h until CsCO_3_ and OA completely reacted, resulting in the formation of a Cs precursor. In parallel, another 100 mL three-necked bottle contained 45 mL of ODE, 166.5 mg of PbBr_2_, and 313.8 mg of PbI_2_. The bottle was injected with 8.4 mL of oleylamine (OAm) and 4.2 mL of OA, and the mixture was dehydrated under vacuum conditions at 120 °C for 1 h. The mixture was then transferred to the fluidic system, namely, the flow system of the Vaportec RS-300 model with R2S pump module (Vaportec Co., Ltd.) for the synthesis of CsPb(Br_0.4_I_0.6_)_3_ PQDs. The as-formed CsPb(Br_0.4_I_0.6_)_3_ PQDs were purified through centrifugation and redispersed in 6 mL of hexane (corresponding to each 25 mL of the initial crude perovskite solution) for storage of CsPb(Br_0.4_I_0.6_)_3_.

#### Synthesis of CsPbBr_3_

Cesium lead bromide perovskite quantum dots (CsPbBr_3_ PQDs) were synthesized using a microfluidic system. The Cs-oleate precursor was synthesized by combining Cs_2_CO_3_ (255 mg), ODE (50 mL), and OA (0.815 mL) and stirring them under vacuum at 120 °C for 1 h to remove unexpected water from the solvent. The resulting composite was cooled to RT and placed under vacuum for the subsequent stage in the microfluidic system. Concurrent with the synthesis of the Cs precursor, the Pb precursor was generated by blending PbBr_2_ (672.3 mg), ZnBr_2_ (413.85 mg), OA (7.5 mL), OAm (7.5 mL), and ODE (50 mL) in a separate container. PbBr_2_ was thoroughly dissolved by stirring the prepared mixture under a vacuum at 120 °C for 1 h. The solution was subsequently stirred under vacuum for the next stage by using the microfluidic system. The microfluidic machine was operated for the synthesis of CsPbBr_3_ PQDs as previously described.^42^ The formed CsPbBr_3_ PQDs were purified through centrifugation and redispersed in 6 mL of hexane (corresponding to each 25 mL of the initial crude perovskite solution) for storage.

The CsPbBr_3_ PQDs were further taken for ligand exchange to obtain products with a high quantum yield by using lecithin soybean. In detail, 15 mg of lecithin soybean was introduced into 1 mL of the cleaned CsPbBr_3_ PQDs. The mixture was then stirred at RT. After 20 min, the as-formed mixture was purified via centrifugation to remove the bigger PQDs. The supernatant CsPbBr_3_ PQDs were obtained for further experiments.

### Hybrid Film Fabrication (HP: Silicone/PMMA)

#### CsPb(Br_0.4_I_0.6_)_3_@Silicone/PMMA Film (RHP Film)

In brief, 1.8 mL of CsPb(Br_0.4_I_0.6_)_3_ PQDs were placed in a Teflon cup. The PQDs-containing cup was placed in the desiccator for 5 min to remove the solvent from the PQDs. After drying, 60 mg of silicone resin A was added and mixed with the as-dried PQDs. After forming the homogeneous mixture, the silicone resin B (240 mg) was introduced and blended until the uniform composite of CsPb(Br_0.4_I_0.6_)_3_@silicone was obtained. Finally, 1500 mg of PMMA solution (133.33 mg/mL) was rapidly introduced and rigorously mixed into the as-formed composite to fabricate the CsPb(Br_0.4_I_0.6_)_3_@silicone/PMMA mixture. The as-blended mixture was further placed in the desiccator for 4 h to form the RHP film at RT.

#### Green CsPbBr_3_@Silicone/PMMA Film (GHP Film)

GHP film was prepared using the same method as the RHP film, replacing CsPb(Br_0.4_I_0.6_)_3_ PQDs with 2 mL of the green CsPbBr_3_ PQDs.

#### Red KSF@Silicone/PMMA Film (Hybrid KSF Film)

The hybrid KSF film was fabricated using the same method as the RHP film, with the only variation being the inclusion of 16.2 mg of KSF.

#### Green Beta-SiAlON:Eu^2+^@Silicone/PMMA Film (Hybrid Beta-SiAlON:Eu^2+^ Film)

The preparation of the green hybrid beta-SiAlON:Eu^2+^ film followed the identical procedure as the RHP film with the sole substitution of 42 mg of beta-SiAlON:Eu^2+^.

### Single Film Fabrication (SP: PQDs/PMMA)

1.8 mL of CsPb(Br_0.4_I_0.6_)_3_ PQDs were placed in the Teflon cup. The PQDs-containing cup was placed in the desiccator for 5 min to remove the solvent from the PQDs. After drying, 1800 mg of PMMA solution (133.33 mg/mL) was introduced and rigorously mixed with the as-dried PQDs. The as-blended mixture was further placed in the desiccator for 4 h to form the film. The GSP film was prepared using the same method as the RSP film and replaced by 2 mL of the green CsPbBr_3_ PQDs.

### Characterization

The CsPbBr_3_ and CsPb(Br_0.4_I_0.6_)_3_ PQD structures and phases were identified using XRD (Bruker D2, Cu Kα radiation source, λ = 1.5418 Å). The morphology and size of PQDs were observed using TEM (JEOL, Japan) and HRTEM (JEOL-2100F, JEOL, Japan). The PL spectra, PLQY, and blue light absorption of the PQDs and PQDs films were achieved using a FluoroMax-4 spectrophotometer (HORIBA, Japan) at an excitation wavelength of 450 nm. Fourier transform infrared (FT-IR) of the as-obtained PQDs was characterized using an FT-IR spectrometer (PerkinElmer, USA). X-ray photoelectron spectroscopy (XPS) was performed using a PHI 5000 Versa Probe apparatus equipped with an Al Kα X-ray source. XPS spectra of all samples were recorded using Cu foil substrates.

## Data Availability

The data that support the findings of this study are available from the corresponding author upon reasonable request.

## Supporting Information Available

XRD, TEM, PL, FTIR of green films, stability test of red and green films with detailed description, XPS of all materials in detail and XANES and EXAFS of green films, absorption energy of RHP film, optical density of red and green films; tables of characteristics of materials, tables of decay time, table of PL emission and fwhm comparison, and a table of optical density of films (PDF)
Preparation of single and hybrid PQD composites (MP4)

## Abbreviations

PQDs: perovskite quantum dots
PLQY: photoluminescence quantum yield
TEM: transmission electron microscope
PMMA: poly(methyl methacrylate)
LED: light-emitting diode
RT: room temperature
HRTEM: high-resolution TEM
XRD: X-ray diffraction
FTIR: Fourier-transform infrared spectroscopy
XPS: X-ray photoelectron spectroscopy
XANES: X-ray absorption spectroscopy
EXAFS: extended X-ray absorption fine structure
RHP film: red CsPb(Br_0.4_I_0.6_)_3_@silicone/PMMA film (red hybrid film)
RSP film: red CsPb(Br_0.4_I_0.6_)_3_@PMMA film (red single film)
GHP film: green CsPbBr_3_@silicone/PMMA film (green hybrid film)
GSP film: green CsPbBr_3_@PMMA film (green single film)
DFT: density functional theory
