# Supplementary material for: Hybrid-Protected Perovskite Quantum Dot Films with Ultra-High Efficiency and Stability for LED Backlighting
Source: ACS Appl Mater Interfaces. 2024 Nov 19;16(48):66262–72. doi: 10.1021/acsami.4c15012 (PMC11622189; doi:10.1021/acsami.4c15012)
Supplement: Supplementary file 1 — am4c15012_si_001.pdf [file am4c15012_si_001.pdf]

Supporting Information

Hybrid-Protected Perovskite Quantum Dot Films with Ultra-High Efficiency and Stability for LED Backlighting

Loan Thi Ngo[a,b]*, Wen-Tse Huang*[a]*, Hemant Verma*[b,c,d]*, Yen-Huei Lin*[a]*, Ling-Wei Liang*[e]*, Chia-Te Fang*[f]*, Jia-Cheng Chang*[f]*, Wen-Chung Chu*[f]*, Chaochin Su*[e,*]*, Chao-Cheng Kaun*[d,*] *and Ru-Shi Liu*[a *]

[a]Department of Chemistry, National Taiwan University, Taipei 106, Taiwan

[b]Nano Science and Technology Program, Taiwan International Graduate Program, Academia Sinica and National Taiwan University, Academia Road 128, Nankang, Taipei 115, Taiwan

[c]Department of Physics, National Taiwan University, Taipei 106, Taiwan

[d]Research Center for Applied Sciences, Academia Sinica, Academia Road 128, Section 2, Nangang, Taipei 11529, Taiwan

[e]Institute of Organic and Polymeric Materials, National Taipei University of Technology, Taipei 106, Taiwan

[f]Eternal Materials Co., Ltd., Kaohsiung City 821, Taiwan

[*]Corresponding authors: Correspondence and requests for materials should be addressed to Ru-Shi Liu (email: rsliu@ntu.edu.tw), Chao-Cheng Kaun (email: kauncc@gate.sinica.edu.tw), or Chaochin Su (email: f10913@mail.ntut.edu.tw)

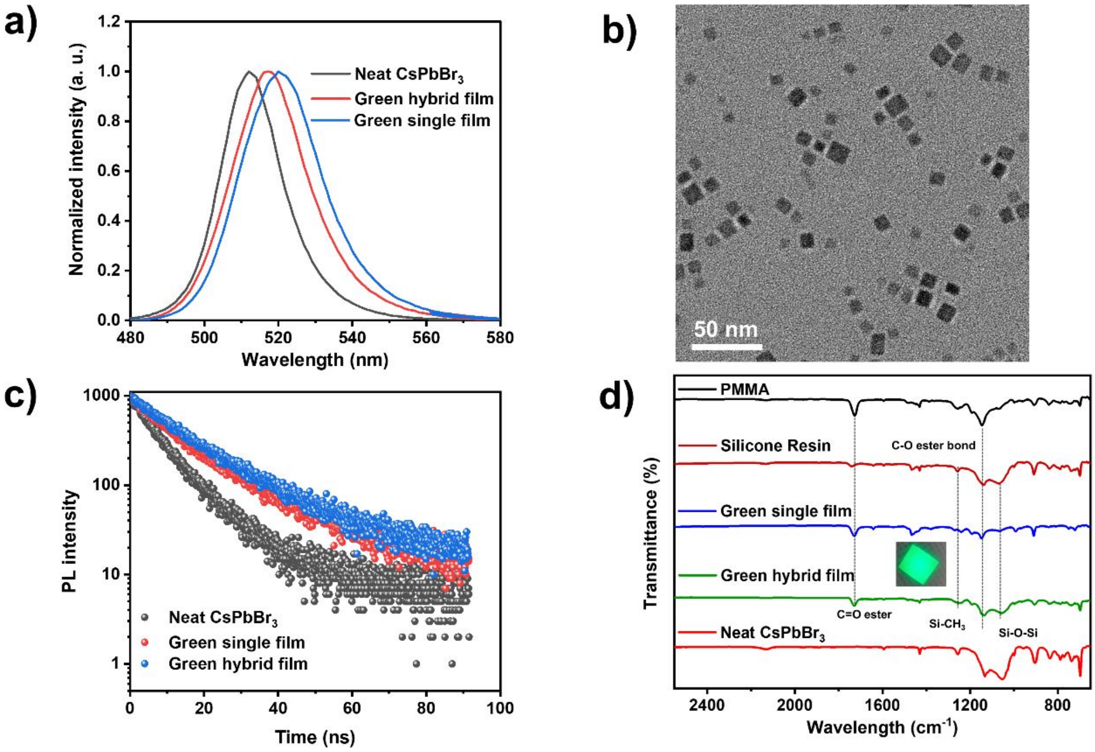

Figure S1. Green PQDs film characterization. (a) PL spectra of neat $CsPbBr_3$ PQDs and green PQD films. (b) TEM images of $CsPbBr_3$ PQDs. (c) Decay curves of neat $CsPbBr_3$ PQDs and green films. (d) FTIR spectra of green PQD films.

The photoluminescence properties of $CsPbBr_3$ PQDs, green targeted $CsPbBr_3$@silicone/PMMA film (GHP film: green hybrid film), and green control $CsPbBr_3$@PMMA film (GSP film: green single film) were investigated using a photoluminescence spectrometer (PL). **Figure S1** shows the characterization of the GHP film. **Figure S1a** shows the normalized PL spectra of the GHP and GSP film with an excitation of 450 nm. Data of emission peaks and full width at half maximum (FWHM) are listed in **Table S1**. From the results of **Figure S1** and **Table S1**, the GHP film has a red shift effect compared with neat $CsPbBr_3$ PQDs. The redshift becomes severe in the GSP film. The phenomenon could be explained by the quantum confinement effect with the change in the size of nanocrystals.

The redshift may have resulted from a slight aggregation of the green PQDs that got into the same one of the films. Moreover, the remarkable QY was observed in the GHP $CsPbBr_3$ film (94.17%), whereas in the film GSP film, the QY was only 74.21%. These results unequivocally illustrate a substantial improvement in the stability and performance of the GHP film compared with $CsPbBr_3$ PQDs and the GSP film.

Figure S1b shows the morphology and particle sizes of the obtained PQDs by using a transmission electron microscope (TEM). The average size of $CsPbBr_3$ PQDs was 11.3 ± 2.4 nm with a cubic shape. We further examined the decay curves of the neat $CsPbBr_3$ PQDs, GHP film, and GSP film.

The decay curves of materials synthesized in our study can be effectively modeled using a tri-exponential function:[1]

$$y = A_1 e^{\frac{(-x-x_0)}{\tau_1}} + A_2 e^{\frac{(-x-x_0)}{\tau_2}} + A_3 e^{\frac{(-x-x_0)}{\tau_3}} \quad (1)$$

Herein, y represents the time-dependent emission intensity, τ_1, τ_2, and τ_3 denote the lifetimes of distinct decay processes, and A_1, A_2, and A_3 are the coefficients corresponding to these lifetimes.

Subsequently, the average lifetime (τ_{avg}) of the films can be determined using the following formula:[1]

$$\tau_{avg} = \frac{A_1\tau_1^2 + A_2\tau_2^2 + A_3\tau_3^2}{A_1\tau_1 + A_2\tau_2 + A_3\tau_3} \quad (2)$$

Data on the lifetime of the aforementioned materials are listed in **Table S2**. **Figure S1c** and **Table S2** present that the decay lifetime increases after forming the film. In contrast to the GSP film and neat $CsPbBr_3$ PQDs, the GHP film displays elevated an extended PL decay lifetime. This observation indicates enhanced film quality and reduced nonradiative recombination following the incorporation of silicone resin.

Figure S1d shows the Fourier transform infrared spectroscopy (FTIR) of GHP $CsPbBr_3$ film and GSP film. C=O stretching of PMMA at 1731 cm^{-1}. The wide peak spanning from 1150 cm^{-1} to 1000 cm^{-1} can be attributed to the stretching vibration of C–O (ester bond). The peak of Si–O–Si at 1062 cm^{-1} can be obviously observed in the GHP film, while this peak is not seen in the GSP film. The peak at 1261 cm^{-1} belonging to the stretching vibration of $Si–CH_3$ can be found in GHP film and silicone resin.[2] This finding confirms the successful encapsulation of the silicone resin and PMMA polymer in the $CsPbBr_3$ PQDs.

Table S1. Emission, FWHM, and PLQY of neat $CsPbBr_3$ PQDs and green PQD films.

**Materials**	**Emission (nm)**	**FWHM (nm)**	**PLQY (%)**
GSP film	523	19	74.21
GHP film	518	19	94.17
Neat $CsPbBr_3$	512	19	91.6

Table S2. Fitted lifetimes, average lifetimes (τ_{avg}) neat $CsPbBr_3$ PQDs, GSP film, and GHP film. A triple-exponential function fitted all decay curves.

**Materials**	**A1**	**τ_1 (ns)**	**A2**	**τ_2 (ns)**	**A3**	**τ_3 (ns)**	**τ_{avg} (ns)**
Neat $CsPbBr_3$	289.8	8.8	673.92	17.73	64.7	247.19	15.00
GSP film	188.03	8.82	403.19	12.80	60.71	145.25	87.07
GHP film	803.63	6.51	0.32	469.41	466.42	9.53	137.32

Table S3. Fitted lifetimes, average lifetimes (τ_{avg}), and PLQY neat $CsPb(Br_{0.4}I_{0.6})_3$ PQDs and RHP film at different ratios of silicone and PMMA.

Silicone : PMMA ratio	A1	τ_1 (ns)	A2	τ_2 (ns)	A3	τ_3 (ns)	τ_{avg} (ns)	PLQY (%)
1:2	61.79	0.11	763.67	147.07	123.81	13.87	145.06	10.78
1:3	N/A	N/A	114.51	12.96	1308.37	202.00	200.95	33.50
1:4	213.16	20.39	N/A	N/A	904.83	326.86	322.42	36.18
1:5	67.91	3.26	599.54	502.92	280.13	3.73E+01	486.97	43.21
1:6	317.06	14.53	N/A	N/A	781.16	144.63	139.53	25.9
$CsPb(Br_{0.4}I_{0.6})_3$	N/A	N/A	710.54	84.33	133.26	15.43	82.05	N/A

Table S4: FWHM and PLQY (%) of reported pure-red perovskite films

Perovskite film	Excitation (nm)	FWHM (nm)	PLQY (%)	References
$CsPbI_{3-x}Br_x$/EC	450	32	34.2	[3]
PBAI-$CsPbI_{0.6}Br_{2.4}$/PEOXA	405	34	65	[4]
$CsPbI_{3-x}Br_x$/PVDF-HFP	365	30	45	[5]
$CsPbI_{3-x}Br_x$ LED film	N/A	40	15.3	[6]
$CsPb(Br_{0.4}I_{0.6})_3$/Silicone/PMMA	450	30	43.21	This work

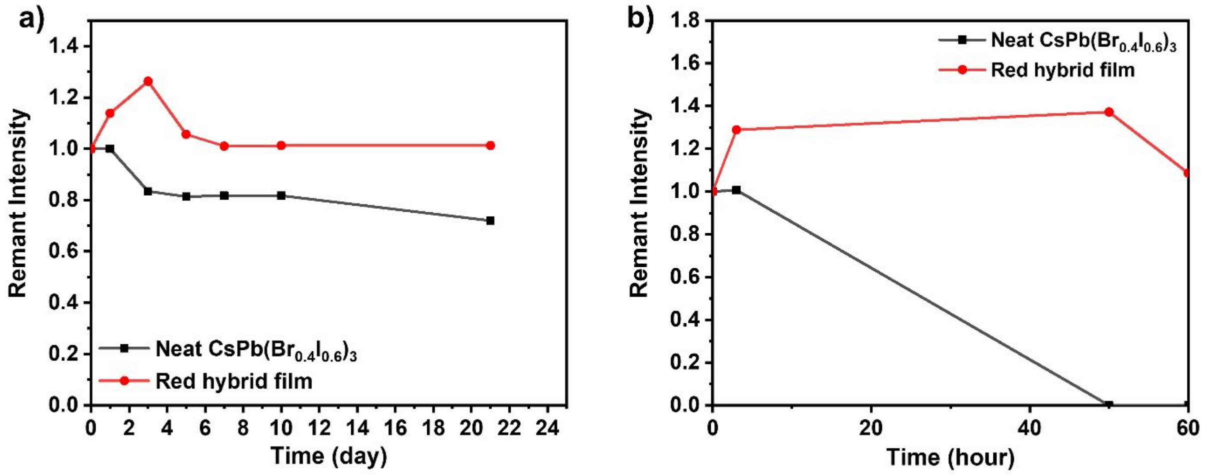

Figure S2. Prolonged stability of the RHP film at (a) air aging and (b) 90°C.

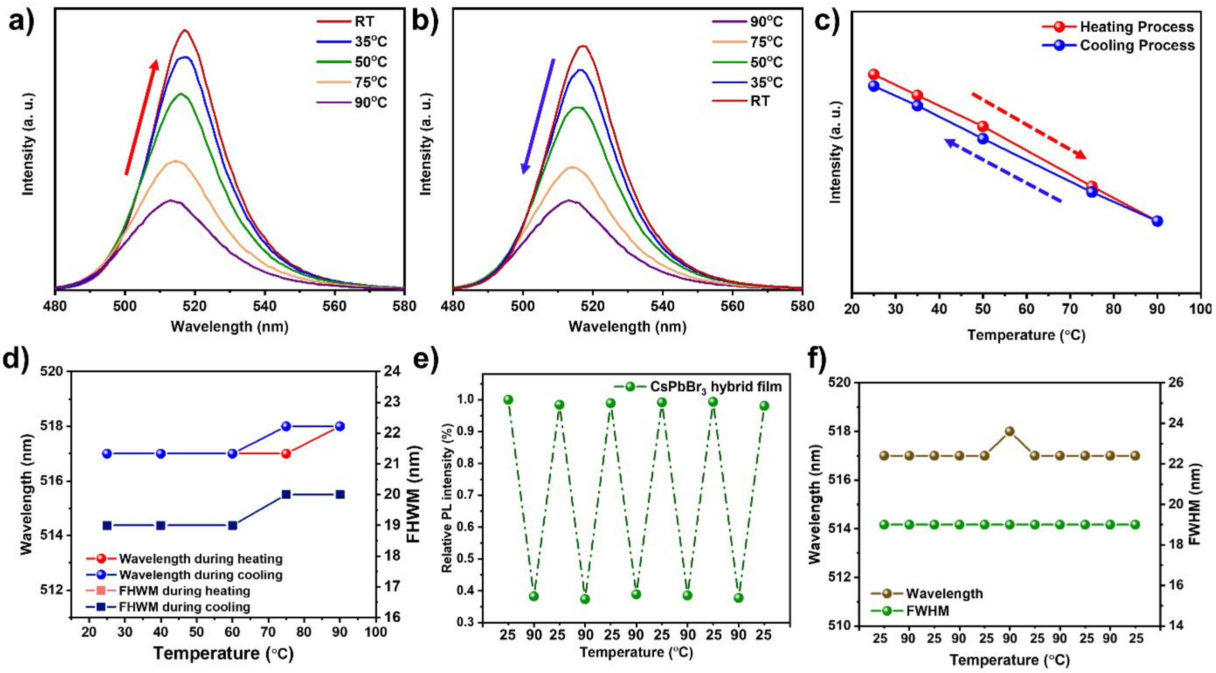

Figure S3. Thermal cycling test. (a) Heating, (b) cooling, comparison of (c) the PL intensity, (d) wavelength and FWHM of GHP film during heating and cooling procedures, (e) PL intensity of the GHP film after undergoing five cycles of heating and cooling between 25°C and 90°C, and (f) the PL emission peaks and FWHM of the GP film after five rounds of heating/cooling processes.

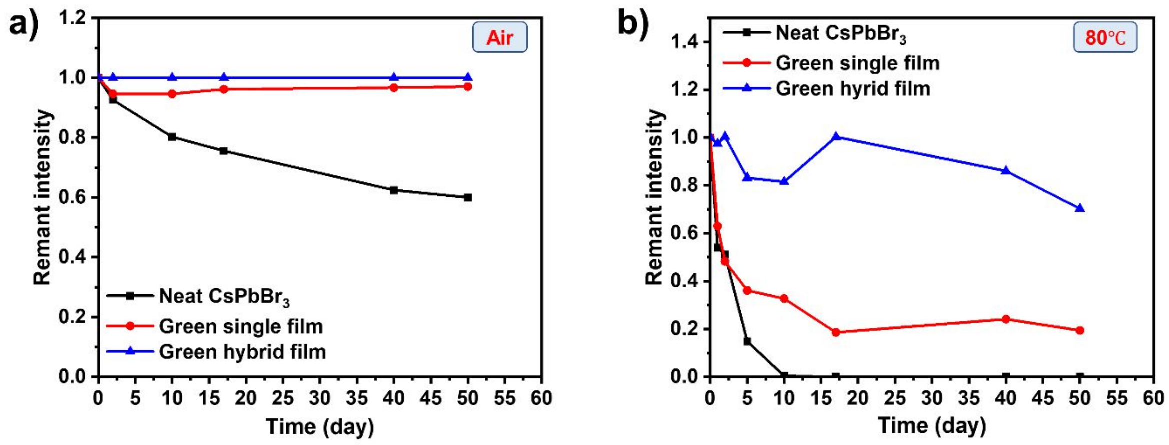

Figure S4. Stability test of the GHP film and GSP film at (a) air aging and (b) 80°C.

The GHP film shows excellent reversibility, approaching 99% of the initial PL intensity after the heating and cooling cycle (**Figure S3a-c**). The change in FWHM and wavelength is negligible (**Figure S3d**). In addition, after five cycles of heating and cooling, the PL intensity of the GHP film is almost unchanged (**Figure S3e**). The FWHM and wavelength of the GHP film are able to return to exact initial values (**Figure S3f**). This implies that our film shows excellent thermal stability. Furthermore, after 50 days of air aging, the PL intensity of the GHP film remains 100% of the initial PL intensity, the GSP film remains 97.05% of the initial one, while the neat $CsPbBr_3$ PQDs only remain about 60% of the initial one (**Figure S4a**). Moreover, the stability of the GHP $CsPbBr_3$ film was further evaluated compared to that of the GSP film. The thermal test at 80°C and 100% moisture resistance were also carried out. The significant difference between the targeted GHP film, the control GSP film, and the neat $CsPbBr_3$ PQDs is also seen after heating them at 80°C. The GHP film remained at 100% of the initial value of PL after 17 days, the GSP film remained at only 18.57% of the initial one after 17 days, while the neat $CsPbBr_3$ PQDs completely lost the PL intensity after 17 days. In addition, after 50 days, the GHP film remains 70.29% of the initial (**Figure S4b**). The performance degradation in luminescence occurs at temperatures exceeding 80°C for the GSP film, which is likely attributed to the glass transition temperature of the PMMA polymer (105°C).[7] However, this

drawback can be overcome after the introduction of silicone resin into the $CsPbBr_3$@PMMA. This finding shows that with the support of silicone in the PQDs films, a significant enhancement in heat is guaranteed.

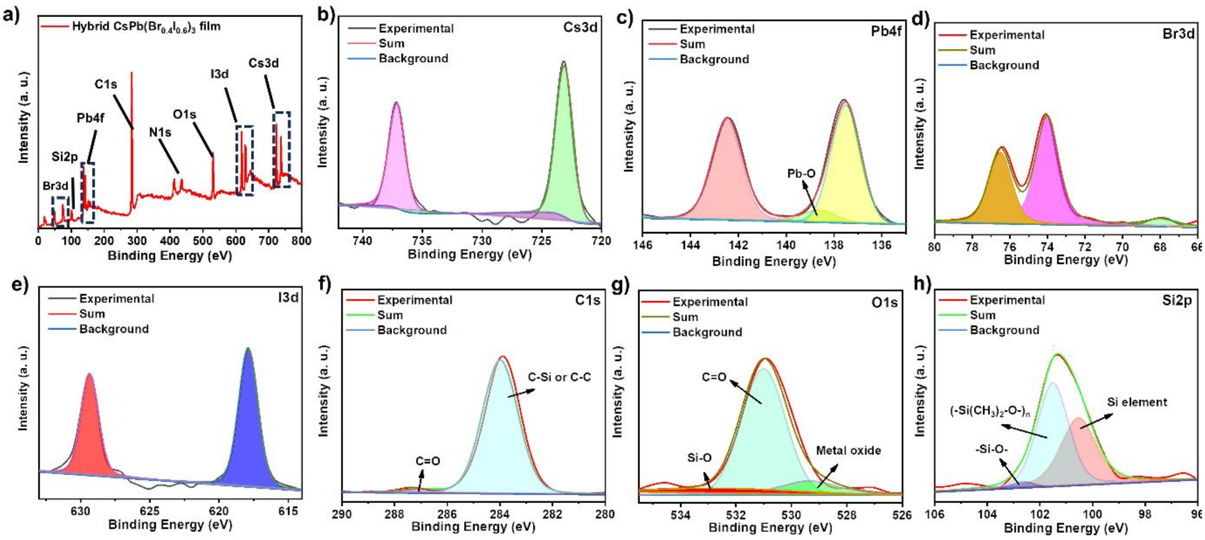

Figure S5. (a) Element survey XPS spectra of the RHP film (b) Cs3d, (c) Pb4f, (d) Br3d, (e) I3d, (f) C1s, (g) O1s, and (h) Si2p XPS spectra of RHP film.

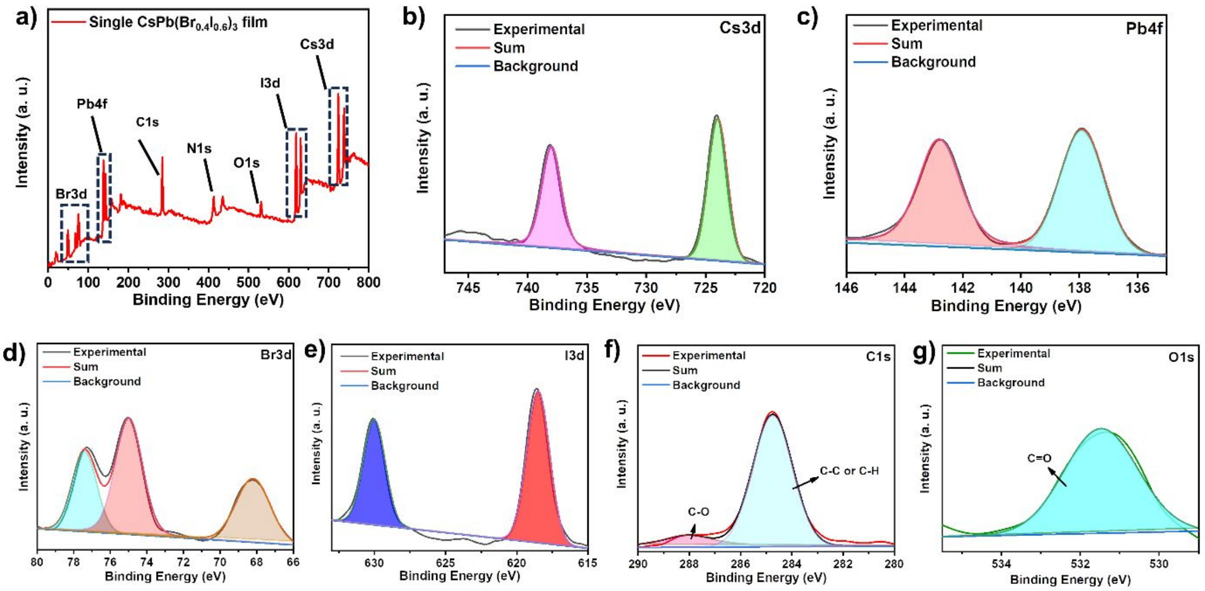

Figure S6. (a) Element survey XPS spectra of RSP film (b) Cs3d, (c) Pb4f, (d) Br3d, (e) I3d, (f) C1s, and (g) O1s spectra of RSP film.

Figures S5a and **S6a** show the element survey XPS spectra of the RHP film and the RSP film, respectively. The new presence of Si2p in the RHP film compared with the RSP film is because of the existence of silicone ingredients in the film. **Figure S5b–h** show the high-resolution Cs3d, Pb4f, Br3d, I3d, C1s, O1s, and Si2p XPS spectra of the RHP film, while the high-resolution Cs3d, Pb4f, Br3d, I3d, C1s, and O1s XPS spectra of the RSP film exhibits in **Figure S6b–g**. The binding energy peaks at 737.1 and 724.2 eV are assigned to $Cs3d_{3/2}$ and $Cs3d_{5/2}$ states, respectively (**Figure S5b**). The peaks at 142.5 and 137.2 eV represent the binding energy states of $Pb4f_{3/2}$ and $Pb4f_{5/2}$, respectively (**Figure S5c**). The signatures centered at 76.2 eV, 74 eV, and 68 eV in **Figure S5d** are attributed to $Br3d_{1/2}$, $Br3d_{3/2,}$ and $Br3d_{3/2}$ states, respectively.[7,8] The 619 eV and 630 eV peaks belong to I3d (**Figure S5e**). All of these peaks are ascribed to the $CsPb(Br_{0.4}I_{0.6})_3$ PQDs.[9] The dual characteristic peaks of C1s observed at 287.4 and 284 eV (**Figure S5f**), along with the O1s peak at 531 eV (**Figure S5g**), align with the binding energy of PMMA.[10,11] In addition, the peaks of Si2p at 101.6 eV ($-Si(CH_3)O-)_n$) and O1s at 532.7 eV (Si–O) are attributed to silicone resin (**Figure S5h**).[12] The binding energy of metal oxide also appears in O1s (529.2 eV) and Pb4f (138.5 eV) (**Figure S5g**). The appearance of these peaks indicates that the combination between $CsPb(Br_{0.4}I_{0.6})_3$ PQDs and silicon resin and PMMA forms Pb–O bond. These peaks are also seen in **Figure 6b–g**. However, some slight shifts can be seen between the GSP film and the GHP film.

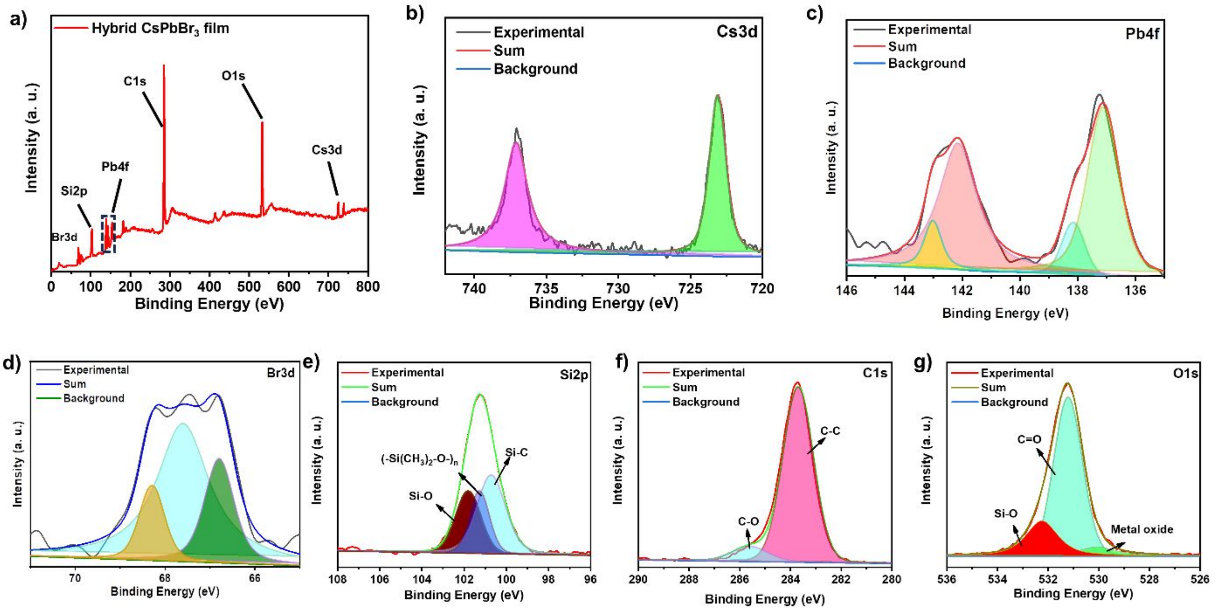

Figure S7. (a) Element survey XPS spectra (b) Cs3d, (c) Pb4f, (d) Br3d, (e) Si2p, (f) C1s, and (g) O1s XPS spectra of GHP $CsPbBr_3$ film.

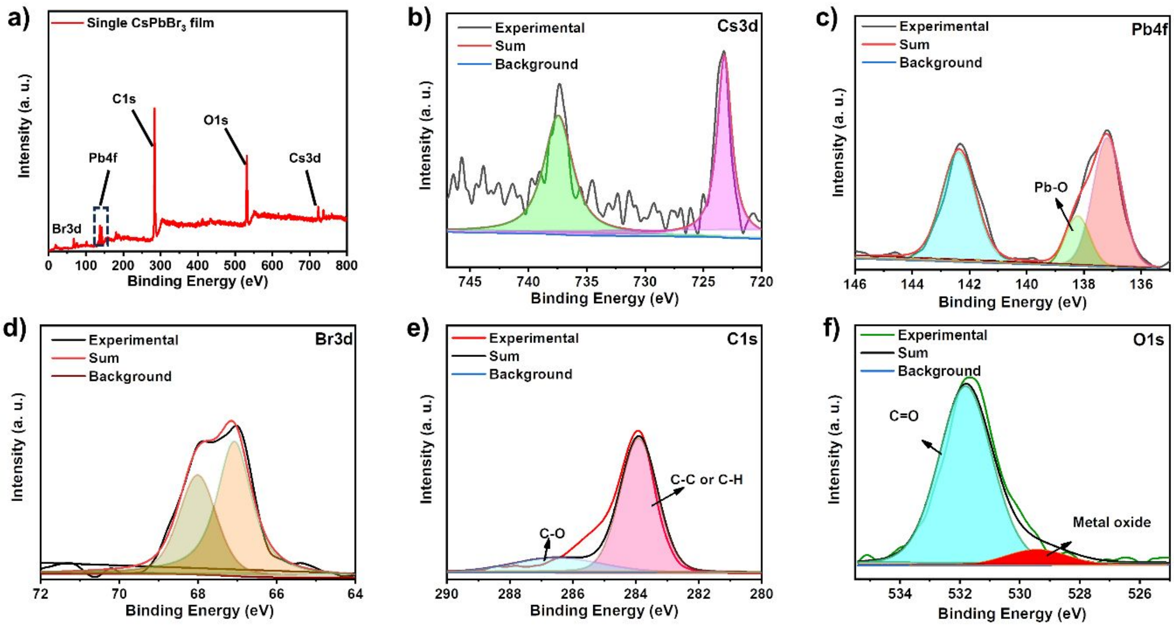

Figure S8. (a) Element survey XPS spectra (b) Cs3d, (c) Pb4f, (d) Br3d, (e) C1s, and (f) O1s XPS spectra of GSP film.

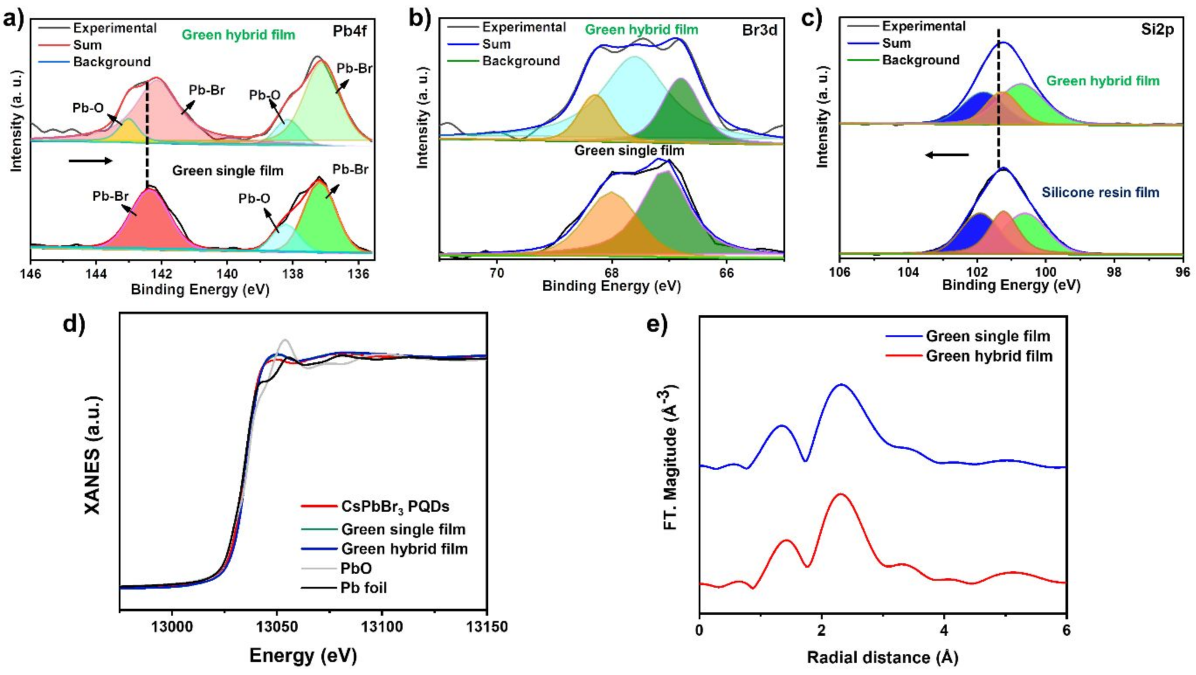

Figure S9. Comparison of XPS spectra of GHP and single films related to (a) Pb4f, (b) Br3d, (c) Si2p, (d) XANES spectra, and (e) Fourier-transformed EXAFS spectra.

Figures S7a and S8a show the element survey XPS spectra of the GHP film and the GSP film, respectively. The presence of Si2p in the GHP film compared with the GSP film is due to the existence of silicone ingredients in the film. **Figures S7b–g** show the high-resolution Cs3d, Pb4f, Br3d, C1s, O1s, and Si2p XPS spectra of the GHP film, while the high-resolution Cs3d, Pb4f, Br3d, C1s, and O1s XPS spectra of the GSP film exhibits in **Figures S8b-f**. The detailed comparison of the two green films is shown in **Figure S9**.

The Pb4f XPS spectrum underwent deconvolution into two doublets in both the GHP film, while the Pb4f XPS of GSP film shows only three peaks elucidating a distinct separation of Pb^{2+} ions with varying chemical environments (**Figure S9a**). These peaks are ascribed to Pb-Br and Pb–O.[13] We postulate that this phenomenon arises from the formation of the Pb-O between the silicone network and PMMA and $CsPbBr_3$, which is well-matched with the phenomenon observed in the red film. This phenomenon can also be seen on the O1s spectrum, which displays an extra shoulder peak at 530.7 eV, indicative of the existence of the O–Pb bond

(**Figures S7g** and **S8f**). The higher the percentage of Pb–O bonds, the higher the decrease in the occurrence of halide vacancy sites surrounding the uncoordinated Pb atoms. Moreover, the noticeable change in the Br3d XPS spectrum, the GHP film has deconvolution into triplets, whereas the Br3d XPS spectrum in the GSP film is deconvoluted into doublets (**Figure S9b**). The slight shift toward the lower binding energy is also seen in the Si2p (**Figure S9c**). This finding implies the establishment of Si–Br bonds between the perovskite and silicone resin.[14] We further performed X-ray absorption spectroscopy (XANES) measurements and the extended X-ray absorption fine structure (EXAFS) analysis (the Pb L_3-edge) for the confirmation of the differences between the two films. As shown in **Figure S9d**, the oxidation state of the two films is at the same position with a minor shift compared with the neat $CsPbBr_3$ PQDs. This phenomenon can arise from changes in the surrounding environment caused by the interaction of polymers with PQDs. Furthermore, the intensity of the Pb–Br bond is higher in the GHP film than in the single film because of the higher Pb–Br bond length disorder in the GSP film compared with that in the GHP film.[15] In addition, Pb–O appears in both films, pointing out that Pb-O bonds are formed from the silicone resin and PMMA. The GSP film exhibits a reduction in the average Pb–O and Pb–Br bond lengths compared with the GHP film, suggesting lattice compression.[16] This finding is attributed to the interaction of silicone resin and PMMA with the PQD surface, consistent with XPS results obtained from the GHP film.

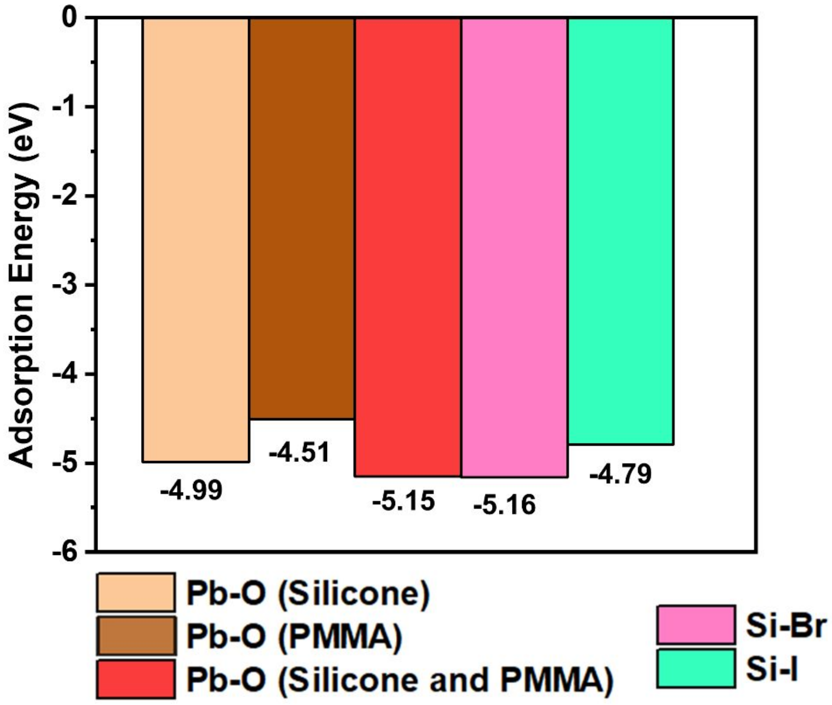

Figure S10. Absorption energy of different absorption sites in the RHP film

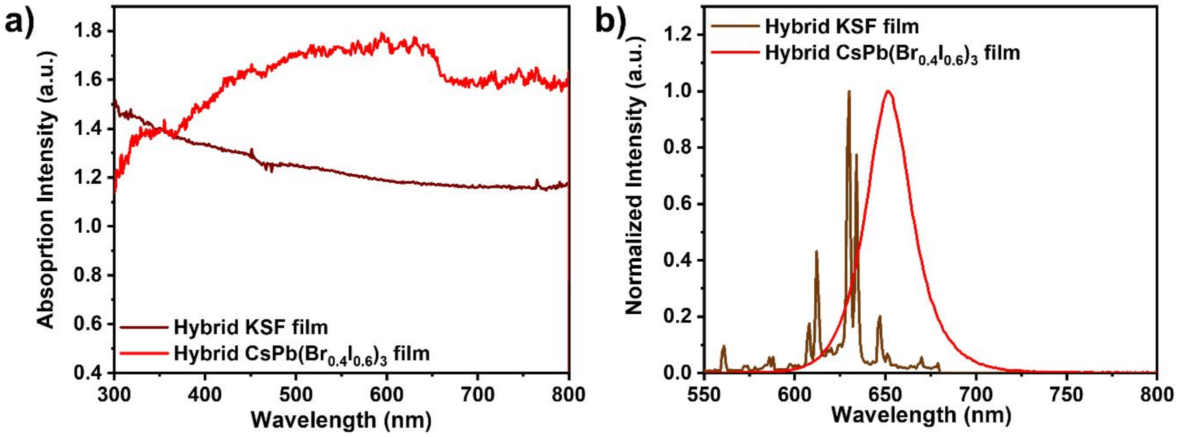

Figure S11. Comparison between RHP film and red hybrid KSF film with the same concentration (0.89%) at 150 μm of thickness. (a) Optical density spectra and (b) PL emission

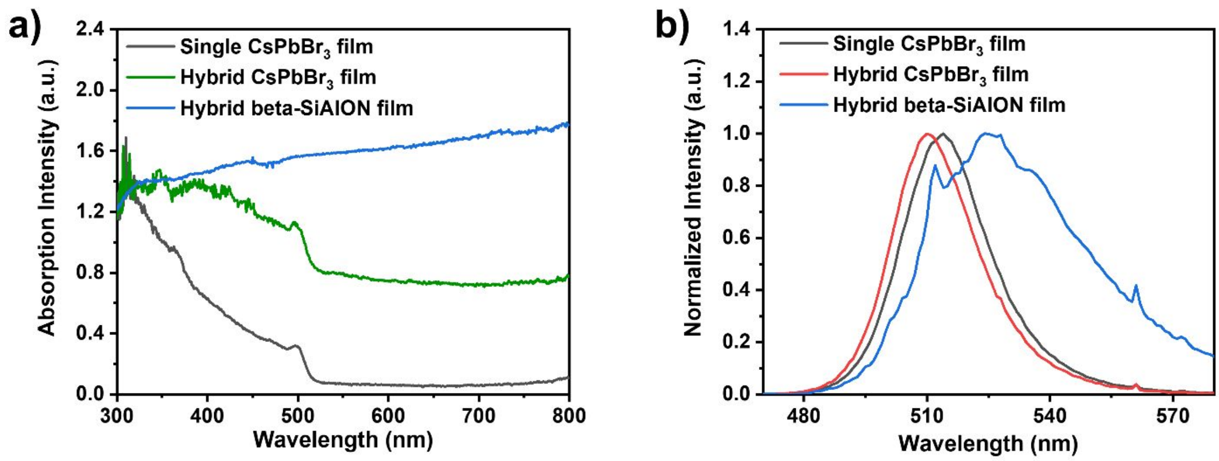

Figure S12. Comparison between green films and green hybrid beta-SiAlON:Eu^{2+} film. (a) Optical density spectra and (b) PL emission.

Table S5. FWHM and PL emission and optical density of green and RHP films, GSP film, and green and red phosphor films.

Perovskite film	FWHM (nm)	Emission	Optical density
RHP film	30	654	1.632
GHP film	24	510	1.228
GSP film	24	514	0.407
Red hybrid KSF film	N/A	Various (from 608 to 647 nm)	1.282
Green hybrid beta-SiAlON:Eu^{2+} film	44	512 and 525	1.519

Figure S12 and **Table S5** show the comparison of blue light absorption with the corresponding emissions of the GHP film, GSP film, and green phosphor beta-SiAlON:Eu^{2+} film (hybrid beta-SiAlON:Eu^{2} film). As shown in **Figure S12a**, with the support of silicone resin, the significant enhancement of the GHP $CsPbBr_3$ film can be seen, excited by emitting

light of about 450 nm. Although a slight blue light absorption difference exists between the GHP film and the green phosphor beta-SiAlON:Eu^{2+} film, the single emission peak with narrower FWHM in the GHP film can be compensated for various emission peaks and large FWHM of the phosphor beta-SiAlON:Eu^{2+} film (**Figure S12b**).

References

(1) Wang, J.; Wang, J.; Li, N.; Du, X.; Ma, J.; He, C.; Li, Z. Direct Z-Scheme 0D/2D Heterojunction of $CsPbBr_3$ Quantum Dots/Bi_2WO_6 Nanosheets for Efficient Photocatalytic CO_2 Reduction. *ACS Appl. Mater. Interfaces* **2020**, *12*, 31477–31485.

(2) Ji, J.; Ge, X.; Pang, X.; Liu, R.; Wen, S.; Sun, J.; Liang, W.; Ge, J.; Chen, X. Synthesis and Characterization of Room Temperature Vulcanized Silicone Rubber Using Methoxyl-Capped MQ Silicone Resin as Self-Reinforced Cross-Linker. *Polymers* **2019**, *11*, 1142.

(3) Song, Y. H.; Choi, S. H.; Yoo, J. S.; Kang, B. K.; Ji, E. K.; Jung, H. S.; Yoon, D. H. Design of Long-term Stable Red-Emitting $CsPb(Br_{0.4}, I_{0.6})_3$ Perovskite Quantum Dot Film for Generation of Warm White Light. *Chem. Eng. J.* **2017**, *313*, 461–465.

(4) Liu, P.; Cai, W.; Zhao, C.; Zhang, S.; Nie, P.; Xu, W.; Meng, H.; Fu, H.; Wei, G. Quasi-2D $CsPbBr_xI_{3-x}$ Composite Thin Films for Efficient and Stable Red Perovskite Light-Emitting Diodes. *Adv. Opt. Mater.* **2021**, *9*, 2101419.

(5) Song, Y. H.; Ge, J.; Mao, L. B.; Wang, K. H.; Tai, X. L.; Zhang, Q.; Tang, L.; Hao, J. M.; Yao, J. S.; Wang, J. J.; Ma, T.; Yang, J. N.; Lan, Y. F.; Ru, X. C.; Feng, L. Z.; Zhang, G.; Lin, Y.; Zhang, Q.; Yao, H. B. Planar Defect–Free Pure Red Perovskite Light-Emitting Diodes via Metastable Phase Crystallization. *Sci. Adv.* **2022**, *8*, eabq2321.

(6) Jiang, M.; Hu, Z.; Ono, L. K.; Qi, Y. $CsPbBr_xI_{3-x}$ Thin Films with Multiple Ammonium Ligands for Low Turn-on Pure-Red Perovskite Light-Emitting Diodes. *Nano Res.* **2021**, *14*, 191–197.

(7) Wang, Z.; Fu, R.; Li, F.; Xie, H.; He, P.; Sha, Q.; Tang, Z.; Wang, N.; Zhong, H. One-Step Polymeric Melt Encapsulation Method to Prepare $CsPbBr_3$ Perovskite Quantum Dots/Polymethyl Methacrylate Composite with High Performance. *Adv. Funct. Mater.* **2021**, *31*, 2010009.

(8) Zhang, M.; Tian, Z. Q.; Zhu, D. L.; He, H.; Guo, S. W.; Chen, Z. L.; Pang, D. W. Stable $CsPbBr_3$ Perovskite Quantum Dots with High Fluorescence Quantum Yields. *New J. Chem.* **2018**, *42*, 9496–9500.

(9) Zhang, D.; Zhao, J.; Liu, Q.; Xia, Z. Synthesis and Luminescence Properties of $CsPbX_3$@Uio-67 Composites toward Stable Photoluminescence Convertors. *Inorg. Chem.* **2019**, *58*, 1690–1696.

(10) Raptis, I.; Kovač, J.; Chatzichristidi, M.; Sarantopoulou, E.; Kollia, Z.; Kobe, S.; Cefalas, A. C. Enhancement of Sensing Properties of Thin Poly(Methyl Methacrylate) Films by VUV Modification. *J. Laser Micro Nanoeng.* **2007**, *2*, 200–205.

(11) Zhu, X. L.; Liu, S. B.; Man, B. Y.; Xie, C. Q.; Chen, D. P.; Wang, D. Q.; Ye, T. C.; Liu, M. Analysis by Using X-Ray Photoelectron Spectroscopy for Polymethyl Methacrylate and Polytetrafluoroethylene Etched by KrF Excimer Laser. *Appl. Surf. Sci.* **2007**, *253*, 3122–3126.

(12) Wang, K.; Chen, M.; Lei, G.; Wang, X. Analysis of Physical–Chemical Properties and Space Environment Adaptability of Two-Component RTV Silicone Rubber. *ACS Omega* **2021**, *6*, 28477–28484.

(13) Jeon, M. G.; Kabir, R. M. D.; Kim, S.; Kirakosyan, A.; Kim, C. Y.; Lee, S. M.; Lee, D. H.; Kim, Y.; Choi, J. Highly Processable and Stable PMMA-Grafted $CsPbBr_3$–SiO_2 Nanoparticles for Down-Conversion Photoluminescence. *Compos. B. Eng.* **2022**, *239*, 109956.

(14) Lin, D.; Xu, X.; Wang, J.; Zhang, T.; Xie, F.; Gong, L.; Chen, J.; Shi, T.; Shi, J.; Liu, P.; Xie, W. Construction of an Iodine Diffusion Barrier Using Network Structure Silicone Resin for Stable Perovskite Solar Cells. *ACS Appl. Mater. Interfaces* **2021**, *13*, 8138–8146.

(15) Singh, H.; Fei, R.; Rakita, Y.; Kulbak, M.; Cahen, D.; Rappe, A. M.; Frenkel, A. I. Origin

of the Anomalous Pb-Br Bond Dynamics in Formamidinium Lead Bromide Perovskites. *Phys. Rev. B* **2020**, *101*, 054302.

(16) Chakraborty, S.; Dash, G.; Mannar, S.; Maurya, K. C.; Das, A.; Narasimhan, S.; Saha, B.; Viswanatha, R. Nonresonant Exciton–Plasmon Interaction in Metal–Chalcogenide (Cu_xS)/Perovskite ($CsPbBr_3$) Based Colloidal Heterostructure. *J. Phys. Chem. C* **2023**, *127*, 15353–15362.
